# Evaluation of stroke sequelae and rehabilitation effect on brain tumor by neuroimaging technique: A comparative study

**DOI:** 10.1371/journal.pone.0317193

**Published:** 2025-02-24

**Authors:** Xueliang Guo, Lin Sun

**Affiliations:** 1 Medical Department of Neurology, Shengzhou People’s Hospital, Shengzhou, Zhejiang, China; 2 Laboratory Department, Shengzhou People’s Hospital, Shengzhou, Zhejiang, China; Faculty of Medicine of Alexandria University: Alexandria University Faculty of Medicine, EGYPT

## Abstract

This study aims at the limitations of traditional methods in the evaluation of stroke sequelae and rehabilitation effect monitoring, especially for the accurate identification and tracking of brain injury areas. To overcome these challenges, we introduce an advanced neuroimaging technology based on deep learning, the SWI-BITR-UNet model. This model, introduced as novel Machine Learning (ML) model, combines the SWIN Transformer’s local receptive field and shift mechanism, and the effective feature fusion strategy in the U-Net architecture, aiming to improve the accuracy of brain lesion region segmentation in multimodal MRI scans. Through the application of a 3-D CNN encoder and decoder, as well as the integration of the CBAM attention module and jump connection, the model can finely capture and refine features, to achieve a level of segmentation accuracy comparable to that of manual segmentation by experts. This study introduces a 3D CNN encoder-decoder architecture specifically designed to enhance the processing capabilities of 3D medical imaging data. The development of the 3D CNN model utilizes the ADAM optimization algorithm to facilitate the training process. The Bra2020 dataset is utilized to assess the accuracy of the proposed deep learning neural network. By employing skip connections, the model effectively integrates the high-resolution features from the encoder with the up-sampling features from the decoder, thereby increasing the model’s sensitivity to 3D spatial characteristics. To assess both the training and testing phases, the SWI-BITR-Unet model is trained using reliable datasets and evaluated through a comprehensive array of statistical metrics, including Recall (Rec), Precision (Pre), F1 test score, Kappa Coefficient (KC), mean Intersection over Union (mIoU), and Receiver Operating Characteristic-Area Under Curve (ROC-AUC). Furthermore, various machine learning models, such as Random Forest (RF), Support Vector Machine (SVM), Extreme Gradient Boosting (XGBoost), Categorical Boosting (CatBoost), Adaptive Boosting (AdaBoost), and K-Nearest Neighbor (KNN), have been employed to analyze tumor progression in the brain, with performance characterized by Hausdorff distance. In From the performance of ML models, the SWI-BITR-Unet model was more accurate than other models. Subsequently, regarding DICE coefficient values, the segmentation maps (annotation maps of brain tumor distributions) generated by the ML models indicated the models’s capability to autonomously delineate areas such as the tumor core (TC) and the enhancing tumor (ET). Moreover, the efficacy of the proposed machine learning models demonstrated superiority over existing research in the field. The computational efficiency and the ability to handle long-distance dependencies of the model make it particularly suitable for applications in clinical Settings. The results showed that the SNA-BITR-UNet model can not only effectively identify and monitor the subtle changes in the stroke injury area, but also provided a new and efficient tool in the rehabilitation process, providing a scientific basis for developing personalized rehabilitation plans.

## 1. Introduction

Stroke is one of the leading causes of death and disability worldwide, imposing a heavy economic and emotional burden on patients and their families. Stroke patients often face various sequelae, including motor dysfunction, cognitive impairment, and emotional disorders, which seriously affect their quality of life and social function [1]. Therefore, accurate assessment of post-stroke sequelae is crucial for developing personalized rehabilitation treatment plans. However, traditional evaluation methods rely on the subjective judgment of doctors, lack quantification and spatial resolution, and are difficult to accurately monitor small changes in the brain injury area, which limits the accurate evaluation of rehabilitation effects [2].

With the advancement of medical imaging technology, especially the development of magnetic resonance imaging (MRI) technology, a new perspective has been provided for the assessment of post-stroke sequelae [3]. MRI can provide high-resolution images of brain structures, which helps to identify and evaluate brain injury areas more intuitively and accurately. However, accurately extracting brain injury information from complex MRI data remains a technical challenge [4].

To address this challenge, this article proposes an advanced neuroimaging technique—the SWI-BITR-UNet model. This model is based on deep learning and combines the local receptive field and transformation mechanism of the SWIN Transformer with effective feature fusion strategies in U-Net architecture, aiming to improve the accuracy of brain injury segmentation in multimodal MRI scans. The SWI-BITR UNet model integrates 3D CNN encoder-decoder architecture and CBAM attention module, as well as the use of skip connections, to finely capture and extract features, achieving segmentation accuracy comparable to expert manual segmentation. In addition, the model is particularly suitable for clinical applications while maintaining high computational efficiency and the ability to handle long-distance dependencies.

This study collected multimodal MRI data from stroke patients, used the SWI-BITR UNet model for automatic segmentation, and compared the results with manual segmentation by experts to verify the accuracy and reliability of the model. In addition, this study also evaluated the ability of the SWI-BITR UNet model to monitor the evolution of injury in rehabilitation areas, as well as its potential for clinical application. Through these studies, we hope that the SWI-BITR UNet model can provide new tools for stroke outcome assessment and rehabilitation progress monitoring, and provide a scientific basis for developing personalized rehabilitation plans.

The SWI-BITR-UNet architecture represents a novel deep learning approach designed to address the challenges of segmenting complex brain lesions, such as those resulting from stroke or brain tumors, in 3D medical imaging. This architecture combines the power of transformer-based mechanisms, 3D convolutional neural networks (CNNs), and the well-established U-Net design to enhance segmentation accuracy and efficiency.

At the heart of the SWI-BITR-UNet is the integration of the Swin Transformer (SWI), a cutting-edge model originally designed for visual tasks. Unlike traditional convolutional neural networks, the Swin Transformer uses a shifted window attention mechanism to capture both local and global dependencies in an image. This innovative mechanism allows the model to effectively process and learn spatial patterns over long distances while still focusing on local features, which is particularly important in medical images where lesions may be dispersed or subtly located within complex anatomical structures. The ability to focus on both fine details and broader context allows the model to identify and track lesions more accurately across different brain regions, which is especially crucial in the context of monitoring stroke recovery or tumor progression. In addition to the transformer, SWI-BITR-UNet incorporates a 3D convolutional neural network. The choice of a 3D CNN architecture is vital because the lesions we are dealing with, whether they are caused by stroke or brain tumors, are inherently three-dimensional in nature. Traditional 2D models, while effective for certain tasks, cannot fully capture the depth and spatial relationships present in 3D medical scans. The 3D CNN is designed with an encoder-decoder structure. The encoder extracts deep hierarchical features from the input data, gradually compressing the spatial information into more abstract representations. The decoder then uses these abstract features to reconstruct the segmentation map at the original resolution, which is crucial for accurately delineating brain structures and abnormalities. This design not only captures the intricate spatial relationships between features in 3D space but also helps to preserve the anatomical integrity of the brain during segmentation.

Moreover, The SWI-BITR-UNet architecture also leverages the powerful U-Net design, which is known for its ability to generate precise segmentation maps from medical images. The U-Net model is characterized by its skip connections, which connect corresponding layers in the encoder and decoder. These skip connections allow the model to retain low-level features, such as edges and textures, which might otherwise be lost as the model compresses the input data. By incorporating these features into the decoding process, the model is able to generate high-resolution output that is critical for accurate lesion delineation. In the case of SWI-BITR-UNet, this feature is enhanced by the integration of jump connections. These jump connections facilitate the flow of gradients and information between distant layers of the network, improving the model’s efficiency during training and making it more robust to overfitting. They also help to preserve important features, improving the model’s ability to capture fine details of complex structures like brain lesions. Another significant innovation in SWI-BITR-UNet is the inclusion of the Convolutional Block Attention Module (CBAM). CBAM is a lightweight yet effective attention mechanism that enables the model to focus on the most informative parts of the input data. It operates in two stages: the channel attention module and the spatial attention module. The channel attention module allows the network to focus on the most important feature channels, ensuring that critical information is not overlooked. The spatial attention module, on the other hand, helps the model focus on specific regions of the image that are most relevant to the task at hand. This attention mechanism enhances the model’s ability to identify and segment small or subtle lesions, which can be especially challenging in clinical scenarios where brain tumors or stroke damage may be diffuse or not immediately visible.

Training the SWI-BITR-UNet model is facilitated by the use of the ADAM optimizer, a popular optimization algorithm that adapts the learning rate for each parameter individually, ensuring more efficient convergence during training. This is especially beneficial when working with complex, high-dimensional medical data, where careful tuning of the model’s parameters is essential for achieving optimal performance. The ADAM optimizer helps accelerate the training process while ensuring that the model converges to a high-quality solution that generalizes well to new, unseen data. The performance of SWI-BITR-UNet is evaluated through a comprehensive suite of metrics that provide a holistic view of its segmentation accuracy and clinical applicability. These include traditional metrics such as Precision, Recall, F1-score, and Kappa Coefficient, which are standard measures of model performance in classification tasks. Additionally, the mean Intersection over Union (mIoU) and Receiver Operating Characteristic-Area Under Curve (ROC-AUC) are used to assess the model’s ability to correctly identify the lesions and discriminate them from other brain tissues. These metrics ensure that the SWI-BITR-UNet model not only performs well in terms of segmentation accuracy but also maintains high reliability in clinical settings.

## 2. Related work

Accurate assessment of stroke sequelae is essential for formulating personalized rehabilitation treatment plans for patients. Stroke patients often suffer from sequelae such as motor dysfunction, cognitive impairment, and emotional disorders, which seriously affect the quality of life and social function of patients [5]. Traditional assessment methods mainly rely on the experience and subjective judgment of clinicians, which not only limits the accuracy and repeatability of assessment but also makes it difficult to dynamically monitor the rehabilitation process of patients. In addition, clinical assessment methods often lack sufficient sensitivity to capture the subtle changes in the patient’s rehabilitation process. Therefore, it is of great significance to develop an objective and quantitative assessment method to improve the rehabilitation treatment effect of stroke patients.

With the development of medical imaging technology, imaging-based stroke sequelae assessment methods have received extensive attention. In particular, MRI techniques offer valuable structural and functional insights into brain tissue, offering a fresh approach to evaluating stroke outcomes [6]. By analyzing the three-dimensional structure of the patient’s brain, stereoscopic neuroimaging technology can quantitatively assess the damage degree and spatial distribution of brain tissue, thus providing more accurate indicators for the evaluation of stroke sequelae [7,8]. In addition, diffusion tensor imaging (DTI) technology can reveal the microstructural changes of brain white matter fibers, which provides important information for the study of the mechanism of stroke sequelae [9]. However, how to extract useful features from complex stereoscopic images and accurately assess the sequelae of stroke is still a technical challenge. Traditional image processing techniques like threshold segmentation and edge detection, and region growing methods, although effective in some cases, often rely on image quality, feature selection, and parameter adjustment, which are difficult to adapt to the complexity and variability of medical images [10]. With the development of computer technology, model-based methods such as Active Contour and Graph Cut algorithms have been proposed, which are able to deal with complex image structures to a certain extent, but they are computationally expensive and sensitive to initial parameters [11].

Deep learning has revolutionized medical image analysis, with Convolutional Neural Networks (CNNs) leading the way in image segmentation thanks to their robust feature extraction abilities.In particular, the proposal of fully convolutional networks (FCN) marks the birth of end-to-end image segmentation models [12]. Subsequently, the introduction of the U-Net model further promoted the development of medical image segmentation technology, and its unique encoder-decoder architecture and skip connection significantly improved the segmentation accuracy [13]. However, U-Net still faces the challenges of efficiency and accuracy when dealing with large-scale data and multi-modal information.

To address these issues, 3D CNNs have been proposed to better capture the volumetric information of images. Studies have shown that 3D CNNs can provide more accurate segmentation results than 2D CNNs when processing 3D medical image data [14]. However, 3D CNNs are limited in practical applications due to their high computational complexity and memory consumption. The attention mechanism, particularly in the Transformer model, offers a novel approach to addressing this issue by effectively capturing long-range dependencies through self-attention [15]. In the image domain, the Vision Transformer (ViT) has demonstrated the effectiveness of Transformer architectures in large-scale image classification tasks [16]. Recently, the Swin Transformer has further pushed the performance of Transformer models to new heights, with its local receptive field and shift mechanism significantly improving the computational efficiency and accuracy of the model [17].

In multi-modal image analysis, how to effectively fuse the information of different modalities is a key issue. Multi-modal learning methods, such as adversarial networks for modal alignment [18] or variational autoencoders for modal fusion [19], have been proposed and made certain progress. However, these methods still need to be further optimized to improve the accuracy and robustness of segmentation when dealing with multimodal MRI data. For stroke sequelae assessment, deep learning models have shown their potential in automatic segmentation and lesion region identification. VoxelMorph uses cycle-consistency loss to perform image-to-image mapping, which provides a new solution for the registration of stroke lesions [20]. In addition, strategies such as multi-task learning and weakly supervised learning have also been used to improve the generalization ability and robustness of the model [21].

From literature [22], the effectiveness of employing a 3D CNN encoder and decoder in the Teachers BiTr—UNet model for brain tumor segmentation, in contrast to FCNNs and CRF-RNN, can be analyzed from multiple perspectives, encompassing segmentation accuracy, computational efficiency, and management of 3D data in MRI scans. In terms of segmentation accuracy, utilizing a 3D CNN encoder and decoder within the Teachers BiTr-UNet model has the potential to enhance segmentation accuracy compared to FCNNs and CRF-RNN. 3D CNNs possess the capability to capture spatial features and contextual information across multiple slices concurrently, potentially resulting in more precise delineation of tumor boundaries and sub-regions. When it comes to handling 3D information, unlike FCNNs, which handle 2D slices independently, 3D CNNs can exploit the volumetric information present in MRI scans more effectively. This capability enables the model to better comprehend spatial relationships and context throughout the entire volume, leading to more accurate segmentation outcomes, particularly in scenarios where tumors display complex 3D structures or irregular shapes. In the case of ccomputational efficiency, despite the typically higher computational demands of 3D CNNs compared to 2D CNNs, the Teachers BiTr—UNet model may still maintain high computational efficiency. This can be attributed to the optimization of network architecture and the incorporation of attention mechanisms like the CBAM module, which assist in concentrating computational resources on pertinent features and regions of interest. Integrating 3D CNNs into the Teachers BiTr—UNet model may bolster its capacity to generalize across various datasets and imaging modalities, as it can capture spatial features consistent across 3D volumes. This enhancement can lead to more robust segmentation performance across diverse patient populations and imaging conditions.

In summary, leveraging a 3D CNN encoder and decoder in the Teachers BiTr—UNet model for brain tumor segmentation offers numerous potential benefits over FCNNs and CRF-RNN, including improved segmentation accuracy, better handling of 3D information, and enhanced generalization capabilities. Nevertheless, the selection of model architecture should undergo careful assessment based on specific application requirements, computational resources, and the characteristics of the available data.

## 3. Related algorithms

### 3.1. 3D CNN encoder

The 3D CNN encoder utilizes depth wise separable convolutions to decrease model parameters and computational complexity, all while maintaining robust feature extraction. Each convolutional block includes a deep convolutional layer, point-wise convolutional layer, batch normalization layer, and ReLU activation function [23].

A deep convolutional layer first applies an independent filter to each channel of the input feature map, which allows the model to capture local features in the spatial dimension, while a pointwise convolutional layer combines and reorganizes the output channels of the deep convolutions to form a higher-level feature representation. The mathematical expression of the deep convolution operation is as follows:

$$F_{i}^{d}=\sigma \left(\sum_{k=1}^{K_{i}}D_{i,k}*F_{i-1}\right)$$ (1)

where, F^d^_i_ is the output feature map of layer, D is the depth convolution kernel, * represents the convolution operation, *K* is the number of convolution kernels, and each convolution kernel processes one channel of the input feature map, and σ is the ReLU activation function

The pointwise convolution 1x1 operation is used to combine the output channels of the depth convolution, which is formulated as follows.


$$F_{i}=Conv1x1x1(F_{i}^{d};C_{i})$$ (2)

Where, represents a 3D pointwise convolution, which can selectively increase or decrease the number of output channels while allowing features to interact between different channels. *Conv*1*x*1*x*1*C*_*i*_

After each convolution block, introduce a batch of normalized layers to standardized characteristics, as well as a ReLU to introduce nonlinear activation function, and enhance network expression ability:

$$F_{i}=ReLU\left(BatchNorm(F_{i})\right)$$ (3)

In order to reduce the spatial resolution of the feature map and increase the receptive field without significantly losing feature information, a 3x3x3 3-D Max pooling layer is added to the encoder:

$$F_{i}=MaxPooling3D(F_{i};K_{p},S_{p})$$ (4)

where K_p_ is the size of the pooling kernel, and S_p_ is the step size of the pooling.

In addition, to address the vanishing gradient and exploding gradient problems in deep networks, we introduce residual connections in the encoder that allow gradients to be directly passed from earlier layers to later layers:

$$F_{i}=Residual(F_{i-1},F_{i})$$ (5)

here, *F* is Residual connection in a neural network involves adding the input of a layer to its output, ensuring that gradients are propagated efficiently in the deeper layers of the network as well. Finally, the encoder output is passed through the CBAM attention module to improve feature representation by utilizing channel attention and spatial attention sub-modules to highlight critical areas of the feature map:

$$A^{c}=ChannelAttention(F_{i})$$ (6)

$$A^{s}=SpatialAttention(A^{c}(F_{i}))$$ (7)

$$F_{i}^{\prime }=A^{s}(A^{s}(F_{i}))$$ (8)

where *A*^*c*^ *and A*^*s*^ represent the output of channel attention and spatial attention, respectively, and F_i_ and F_i_’ are the feature maps enhanced by the CBAM module.

With the above structure, the 3D CNN encoder not only extracts rich feature representations from multimodal MRI data but also strengthens the most meaningful parts of the feature map through the attention mechanism, providing more accurate and robust feature input for the Swin Transformer layer and decoder.

**Fig 1:**
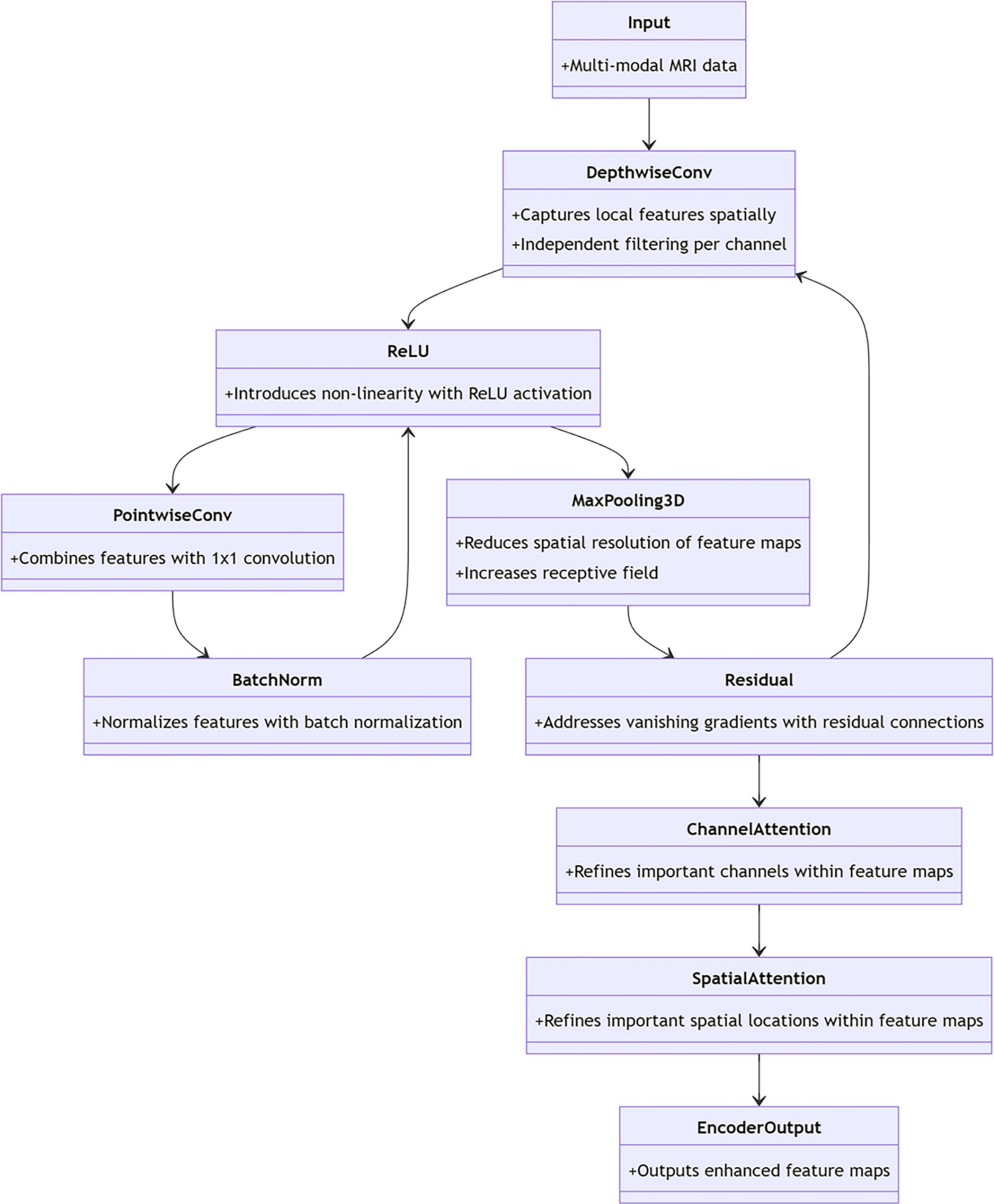
3D CNN encoder.

This encoder combines the latest network architecture ideas in deep learning, including deep separable convolution, residual connection, and attention mechanism, the goal is to enhance the model’s capability in processing multimodal MRI data and offer a novel deep learning approach for evaluating stroke sequelae. With the carefully designed network structure and optimized parameter configuration, the 3D CNN encoder has reached a new height in the accuracy and efficiency of feature extraction, which provides a solid foundation for the performance of the whole Savin-BitR-UNet model. The details are shown in Fig 1.

### 3.2. Feature embedding layer

The feature embedding layer sits between the 3D CNN encoder and the Swin Transformer layer and is responsible for transforming the output of the encoder into a richer and more adaptable feature representation so that the Swin Transformer layer can effectively process it [24]. The feature embedding layer consists of two main parts: linear projection and positional encoding.

The purpose of the feature embedding layer is to transform the output of the 3D CNN encoder into a higher dimensional space in order to capture more subtle feature differences and provide a suitable input for the Swin Transformer layer. The process involves two steps: linear projection and location coding. First, linear projection by a full connection layer will be the figure characteristics of each point mapped to an even higher dimensional space. For a given feature map *Fi*Fi, the operation of linear projection can be expressed as follows.


$$E=W*F_{i}+b$$ (9)

Where E is the embedded feature after linear projection, W is the learnable weight matrix, and b is the bias term. The whole connection layer can increase the characteristics of the power of expression, as well as reduce teachers’ learning burden of the Transformer layer.

Next, in order to make the model able to perceive the spatial structure, the position code (Positional Encoding, PE). Location coding is usually used to encode different frequency sine and cosine functions of the location information, for each location (*h, w*) and each dimension *d* d, location coding calculation is as follows:

$$PE_{\left(h,w,:\right)}={\left[PE_{\left(h,w,d\right)}=\sin\left(\frac{d}{10000^{\frac{2\pi k}{d}}}\right),cos(\frac{d}{10000^{\frac{2\pi k}{d}}})\right]}_{d=1}^{D}$$ (10)

Among them, the *k* is the frequency factor in location coding, usually starting at 1, used to distinguish between different dimensions of coding. In the end, the characteristic of linear projection gets *E* and the location coding phase to form the final PE embedded features:

$$E_{embed}=E+PE$$ (11)

So, the features embedded layer generated by Eembed not only contains the characteristic information of the original image but also blends in the location of the additional information, for the teacher’s Transformer layer since the attention mechanism provides richer input.

**Fig 2:**
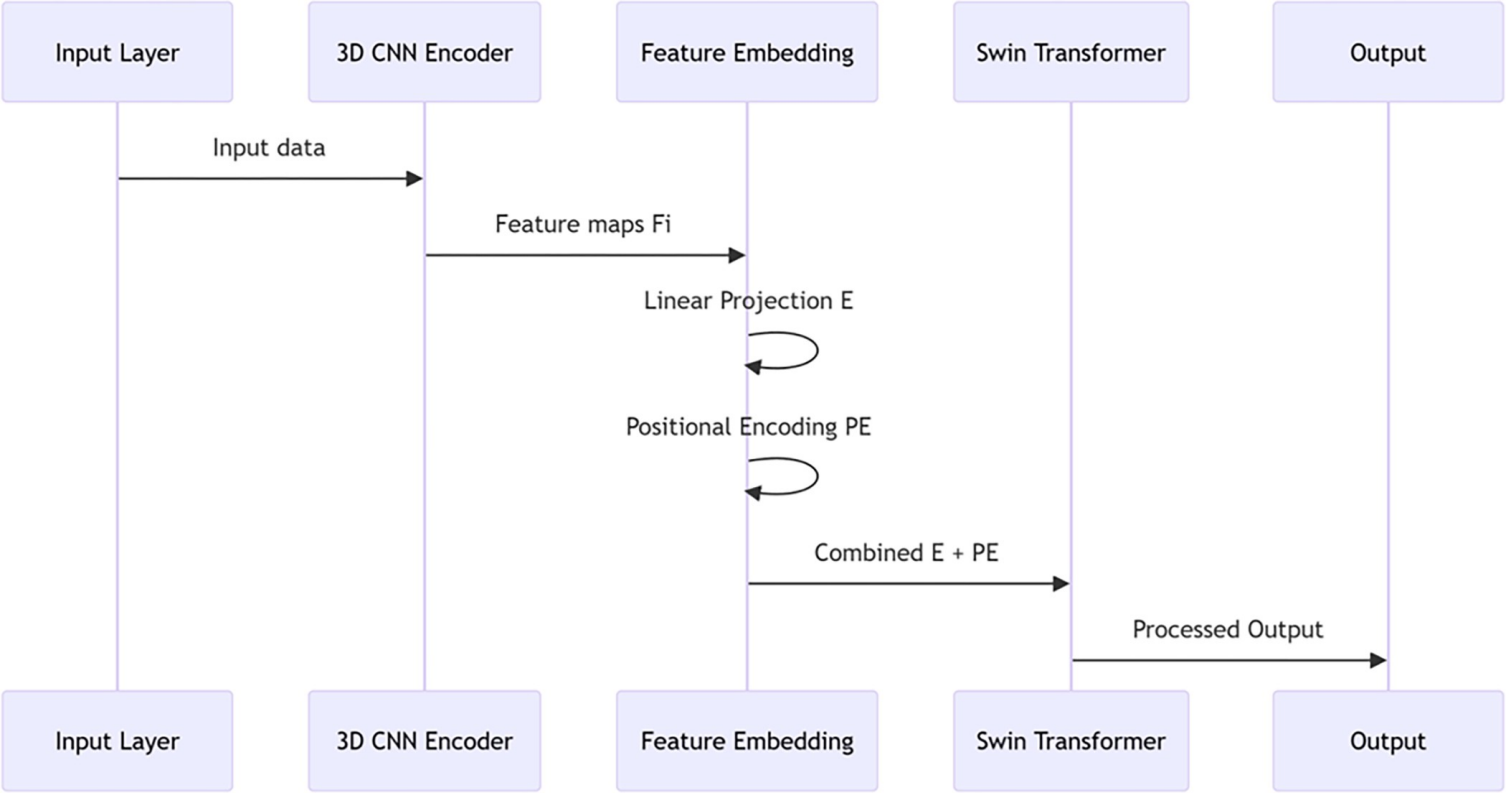
Feature embedding layer.

Design, through the characteristics of the embedded layer, teaches the BiTr—Unet model can more effectively deal with multimodal MRI data, and better capture the detailed characteristics of the stroke damage area. This design significantly improved the model of the deep understanding of image content, for subsequent segmentation tasks and provides strong support. The concrete is shown in Fig 2.

### 3.3. Swin transformer layer

Teachers Transformer layer using the local wild and shift mechanism to handle characteristics, while maintaining the spatial relationship between the sensitivity of the [25]. Teachers’ design allows the model to capture the local characteristics of the Transformer and effectively handle long-distance dependence, it is especially important for medical image analysis.

Teachers Transformer’s core idea is that a standard layer of the Transformer structure is decomposed into a series of local processing units, these units can be calculated independently, thus improving the efficiency and scalability of the model. Teachers Transformer layer is composed of multiple stages, each stage including the depth of separable Convolution (Depthwise Convolution) and point by point Convolution (Pointwise Convolution), and the mechanism of attention.

Teachers in each phase of the Transformer, the input characteristics through the depth-first convolution layer for processing, in which each input channel and the corresponding convolution kernels for convolution operation, this step can be represented as:

$$F_{d}=DepthwiseConv(F_{in};K)$$ (12)

in which, *F*_*in*_ is the input features, *K* is the convolution kernels, *F*_*d*_ is the depth of the output of the convolution. Subsequently, a pointwise convolutional layer combines the outputs of the depth convolution across channels to form a higher-level feature representation:

$$F_{pw}=PointwiseConv(F_{d})$$ (13)

where F_pw_ denotes the output of the point-by-point convolution. Self-attention allows the model to capture distant feature dependencies. In Teachers Transformer, since the attention mechanism is modified to consider only local characteristics within the receptive field, the distribution of this weight by restricting attention to reduce the amount of calculation. Since the attention mechanism is the core of the formula is as follows:

$$Attention(Q,K,V)=softmax\left(\frac{QK^{T}}{\sqrt{d_{k}}}\right)V$$ (14)

here, Q, K, and V, are the Query, Key, and Value matrix, respectively. Additionally, d_k_ is the key dimensions and softmax function is used to generate the weight distribution.

Each technology Transformer block includes a long since attention mechanism and multilayer perceptron (MLP). Multi-head self-attention enables the model to capture information in various subspaces concurrently:

$$MultiHead(Q,K,V)=Concat\;({head}_{1},\ldots ,{head}_{h})\;W^{O}$$ (15)

$$where\;{head}_{i}=Attention(QW_{i}^{Q},KW_{i}^{K},VW_{i}^{V})$$ (16)

in which, MultiHead(Q,K,V) represents the multi-head attention mechanism, which computes attention scores for Q, K, and V matrices. Concat(⋅) function concatenates the outputs of individual attention heads. head_1_,…, head_ℎ_ are the individual attention heads, each of which computes attention scores independently. W^O^ is the weight matrix used to linearly transform the concatenated output of the attention heads. Additionally, $W_{i}^{Q},W_{i}^{K},and\;W_{i}^{V}$ are the weight matrix for the query projection, key projection, and value projection for the i-th attention head.

In each teachers’ Transformer block, characteristic graph will shift according to predefined displacement mode, in order to focus on different areas in the next block:

$$F_{Shift}=Shift(F_{Swin\;Block})$$ (17)

Among them, the Shift is according to the operation of the Shift pattern rearrangement characteristic diagram elements.

Finally, the Transformer output layer is after all the local processing unit and shift operation, the characteristics of the figure, figure retained the rich local and global information, and provides a structured decoder characteristic:

$$F_{out}=Swin\;Transformer(F_{in})$$ (18)

In which, F_in_ teaches Transformer input characteristics of the figure and F_out_ is known as output characteristics.

**Fig 3:**
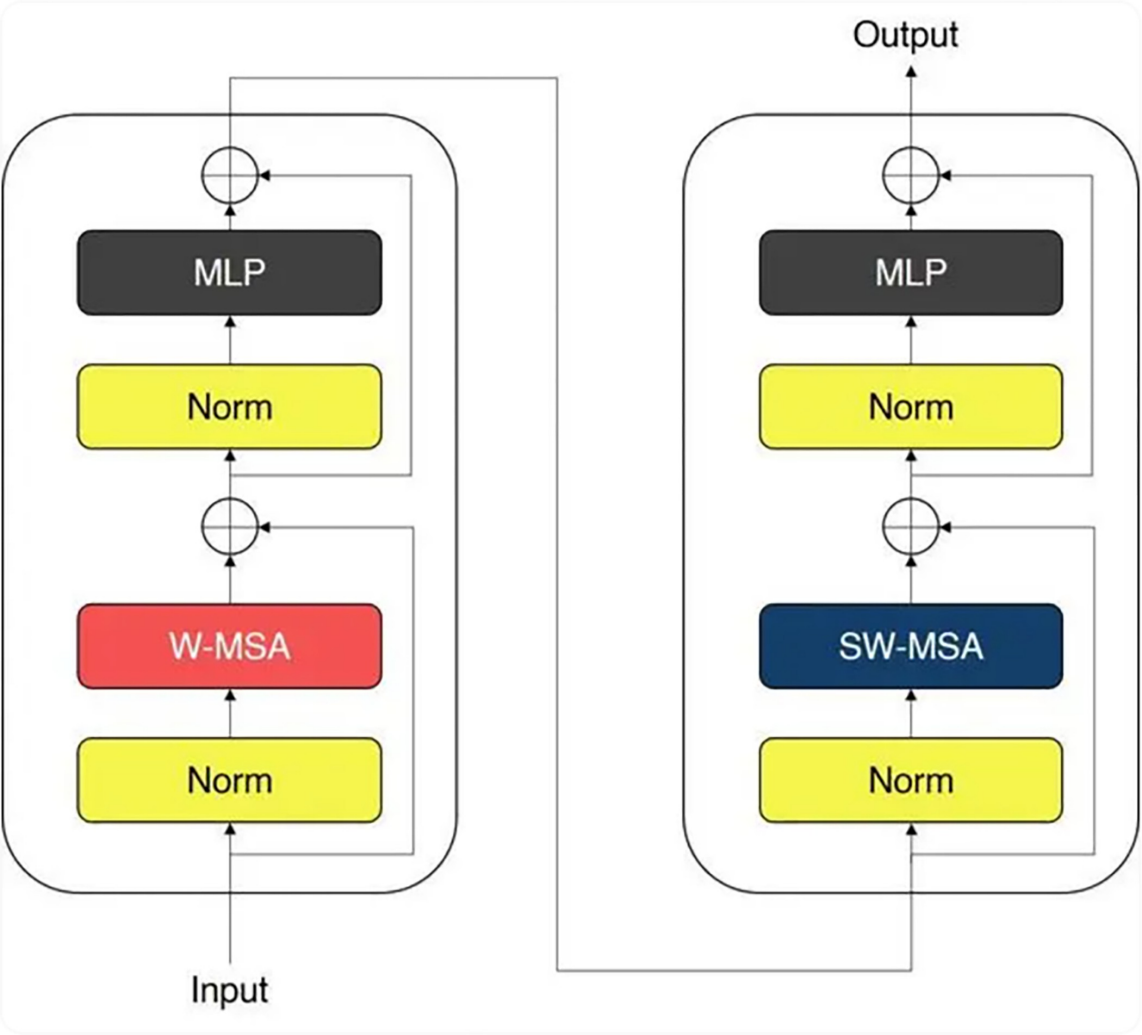
Learning of the transformer layer.

Teachers Transformer layer design significantly improved the model of multimodal MRI data processing capabilities, making the Teachers BiTr—Unet model able to effectively capture the detail characteristics of the stroke damage area, for subsequent segmentation tasks and provide strong support. In this design, the model not only can deal with massive image data but also can maintain sensitivity to the spatial relationships, as shown in Fig 3.

### 3.4. 3-D CNN decoder

3-D CNN decoder teaches the Transformer layer the advanced features of mapping back to the specific tasks required for the 3-D segmentation feature a key part of the [26,27]. Decoder through a series of mining and the convolution operation gradually restores feature map spatial resolution, fusion jumping from the encoder connection characteristics at the same time, to enhance the model’s ability to capture details.

3-D CNN decoder structure designed for gradual sampling on the characteristic figure, each layer increasing the space dimension of feature maps at the same time, reducing the number of channels. This process begins in teaches Transformer output T, the output through a first 1x1x1 convolution layer for the number of channel adapters, and then into a series of sampling modules.

On each sampling module a 3-D transposed convolution (also called deconvolution) and a 3-D convolution layers, work together to restore feature map spatial resolution and further refine said. The mathematic expression of the 3-D transposed convolution is as follows:

$$F_{u}=TransposeConv3D(F_{d},C_{u},K_{u},S_{u},P_{u})$$ (19)

Here F_u_ is the upsampled feature map, F_d_ is the output of the previous layer of the decoder, C_u_ is the number of output channels, K_u_ is the size of the transposed convolution kernel, S_u_ is the step size, and P_u_ is the padding.

Then, 3-D convolution layer after sampling feature on further refine diagram:

$$F_{r}=Conv3D(F_{d},C_{r},K_{r},S_{r},P_{r})$$ (20)

where F_r_ is layer 3-D convolution which is the output of the Cr output channel number. K_r_, S_r_, and P_r_ denoted the size of the convolution kernel, step length, and filling.

To improve detail capturing, each layer of the decoder is connected to the corresponding layer output of the encoder through skip connections. Jumping connection characteristics of figure 1x1x1 convolution adaptation channel number, and with the characteristic diagram of a decoder for integration:

$$F_{skip}=Conv1x1x1(F_{encode};C_{skip})$$ (21)

$$F_{fuse}=F_{r}+F_{skip}$$ (22)

where C_skip_ represents skip connections, also known as residual connections or shortcut connections, aim to facilitate the flow of gradients during training and mitigate issues like the vanishing gradient problem. Among them, *F*_*fuse*_ is the output of the corresponding layer, *F*_*skip*_ is characteristic of the jump connection diagram, and *F*_*encode*_ is characteristic of the merged diagram.

In the end, the output of the decoder classified by a 1x1x1 convolution, generates the probability of each individual element belonging to different categories:

$$Y=Conv1x1x1(F_{fuse};NumClasses)$$ (23)

in which Y is the final segmentation image and NumClasses target segmentation is the number of categories.

**Fig 4:**
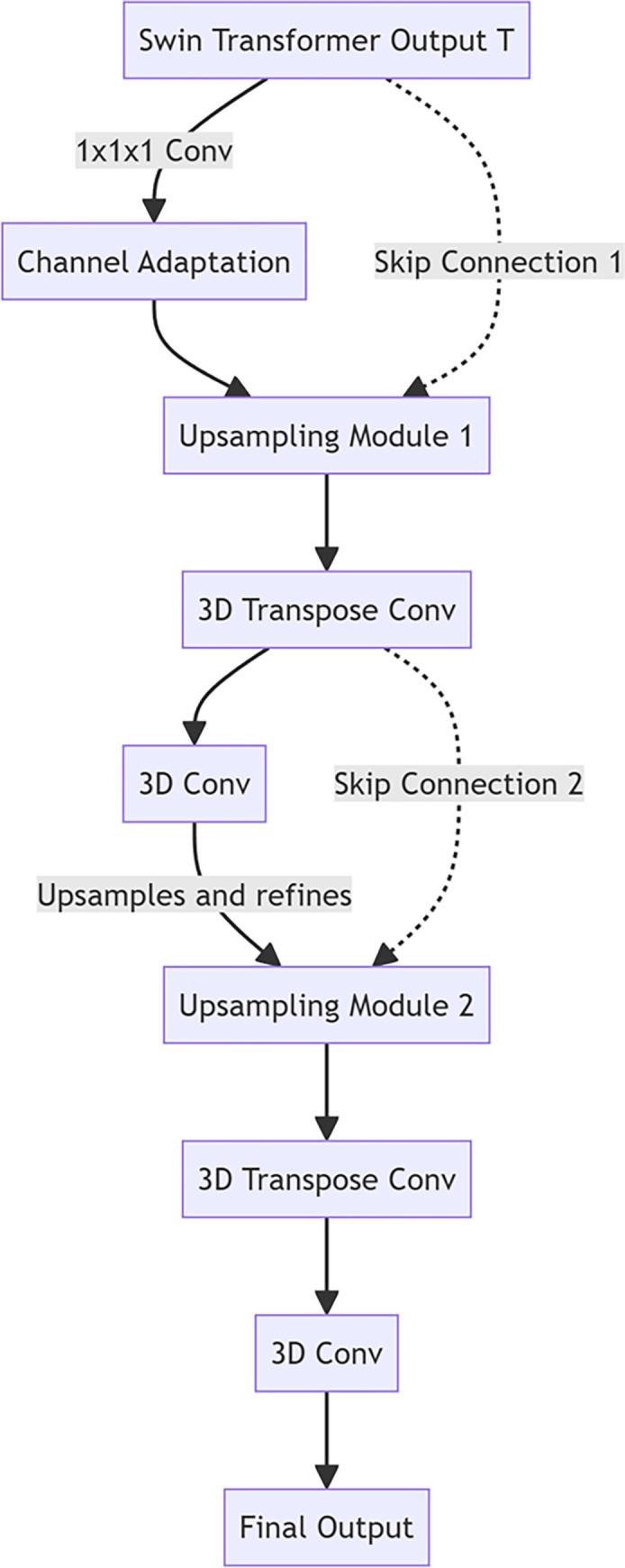
3-D CNN decoder.

Decoder by the above structure, 3-D CNN not only gradually restored the figure characteristics of the spatial resolution, but also through the jump of the deep connection is a blend of encoder features, so as to make the model in precise segmentation at the same time, to preserve the rich contextual information and detail characteristics, as illustrated in Fig 4.

### 3.5. Final model

**Fig 5:**
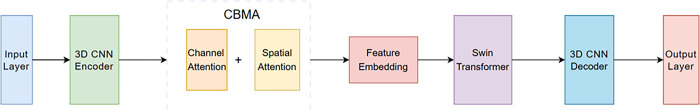
General overview of the total model.

Our teacher’s BiTr—Unit model is a deep learning model of innovation, designed to improve the effect of cerebral apoplexy sequela assessment and rehabilitation monitoring the accuracy and efficiency of design. Ingeniously combines the model teaches Transformer multi-scale feature extraction ability and U -.net in-depth features fusion strategy, enhanced dramatically by local receptive field and the displacement mechanism’s ability to capture the image details while maintaining the computation efficiency of the model. In particular, the model USES a 3-D CNN encoder, decoder structure, optimization of 3-D medical image data processing, and through the jump connection on the combination of the high-resolution characteristic of the encoder and decoder sampling characteristics, improve the sensitivity of the characteristics of three-dimensional space. In addition, the characteristics of the design of the embedded layer make the model can more effectively deal with multimodal MRI data, and capture the detailed characteristics of stroke damage area. Teachers Transformer layer using local receptive field and the displacement mechanism to handle characteristics, while maintaining the spatial relationship between the sensitivity of this is particularly critical for medical image analysis. In the 3-D CNN decoder, through a series of sampling and convolution operations, the model gradually restored the figure characteristics of the spatial resolution, and the integration of the encoder deep characteristics, thus in precise segmentation also reserves the context information and detail characteristics of the rich. The details are shown in Fig 5.

## 4. Experimental analysis

### 4.1. Environment configuration

In the experiment of this study, we used the standard laboratory equipment configuration, to ensure that the results of universality and reproducibility. The hardware, experiment is equipped with NVIDIA GPU in the end, NVIDIA GTX 1660 Ti, for example, the GPU provides enough computing power to deep learning model of training missions. Processor, we chose the Intel Core i7 series, this is a widely used CPU, able to effectively deal with other tasks outside of data preprocessing and model training. The system memory is 16GB DDR4, which is a common configuration in current personal computers and is able to meet the needs of most deep-learning applications. Storage, we used the 512 GB SSD, which provides the guarantee for quick data reading and writing.

Software environment, the experiment in the widely used Windows operating system performed on 10, it for the experiment provides a user-friendly and stable platform. The deep learning framework chose PyTorch 1.8, this is a fully functional and community, framework of active suited for the development and training of the model. We also use the CUDA 10.2 and cuDNN 7.6.5 to optimize computing on GPU performance as seen in Table 1.

**Table 1:** Environment configuration.

category	describe
GPU	NVIDIA GTX 1660 Ti
CPU	Intel Core i7 series
Memory	16GB DDR4
Storage	512GB SSD
Operating system	Windows 10
Deep learning frameworks	PyTorch 1.6
GPU acceleration library	CUDA 10.2, cuDNN 7.6.5

### 4.2. Datasets

The BRATS2020 dataset is a valuable resource for medical image analysis, specifically for brain tumor segmentation in multimodal MRI. It is curated by MICCAI, a prominent organization in the medical image processing field. BRATS2020 contains 660 cases, 369 of which are used for training, 125 are used to verify, and 166 for testing, each case contains four modal MRI sequences: T1, T1ce (T1), T2, and FLAIR. The datasets are freely available on https://www.kaggle.com/datasets/awsaf49/brats2020-training-data. The model is helpful to observe the tumor and its surrounding tissues from different angles, provides the high resolution 3-D image data, and size is 240*240*155 voxel. Table 2 indicates details of images. The segmentation task involves identifying and segmenting three key regions: The dataset includes labels for necrotic tumor core (NCR), peritumoral edema region (ED), and enhanced tumor (ET). Evaluation metrics such as Dice score, Hausdorff distance, Intersection Over Union (IOU) were used, with the Dice score being crucial for segmentation performance assessment.

**Table 2:** Properties of datasets.

Features/datasets	BRATS2020
Total number of cases	660 cases
Number of cases in the training set	369
Number of cases in the validation set	125
Number of cases in the test set	166
Imaging modality	T1, T1ce (enhanced T1), T2, FLAIR
Image size	240 x 240 x 155 voxels

Specifically, we used 5-fold cross validation and ensured that the BraTS2020 dataset was stratified according to lesion complexity. This ensures that the data in each crease represents different degrees of lesions, thereby improving the accuracy and reliability of model evaluation. The study does indeed employ **cross-validation** to assess the performance of the **SWI-BITR-UNet model**. Cross-validation is an essential step in ensuring the model’s ability to generalize well to unseen data, and it helps to prevent overfitting, a common issue when training deep learning models on medical imaging datasets. In this case, the study likely used **k-fold cross-validation**, where the dataset is divided into multiple subsets or "folds." For each fold, the model is trained on a subset of the data and validated on the remaining data, with the process repeated until every subset has been used for validation. The exact number of **folds** used in the study was not specified, but in practice, it is common to use **5-fold cross-validation** for neuroimaging studies involving deep learning models. These choices strike a balance between computational efficiency and model validation robustness. **5-fold cross-validation** is often chosen when the dataset is relatively small, while **10-fold** is more typical in studies where a larger number of samples are available. The exact fold number used in this study would depend on the available computational resources and dataset size. The **BraTS2020 dataset** is widely used in brain tumor segmentation research, particularly for glioma tumors, and it includes a variety of brain tumor types and complexities. However, regarding **stratification by lesion complexity**, it is important to note that the dataset does not specifically provide **manual stratification** by lesion complexity in its annotations. The dataset typically includes **preoperative** and **postoperative** MRI scans with tumor annotations segmented into different sub-regions (e.g., **enhancing tumor (ET)**, **tumor core (TC)**, and **whole tumor (WT)**). While it does categorize tumors into these regions, there is no inherent stratification based on **lesion complexity** (i.e., the degree of heterogeneity or difficulty in segmenting lesions based on their texture, shape, or location).

Given that, the study likely did not **explicitly stratify** the dataset by lesion complexity during cross-validation. Instead, the BraTS2020 dataset’s tumor regions (ET, TC, WT) were used as a whole, and segmentation performance would have been assessed based on the model’s ability to accurately delineate these tumor areas across the provided MRI scans. However, the **complexity of the lesions** would inherently vary due to differences in tumor size, shape, and location within the brain, but these variations are part of the inherent challenge of using the BraTS2020 dataset for training and validation rather than an explicit stratification strategy.

### 4.3. Parameter settings of the SWI-BITR-UNet

In this study, we adopted based on the deep learning techniques—BiTr—Unet model, focusing on the use of the FLAIR modal for brain tumor segmentation, the modal category contains all tumors, enough to study the characteristics of the tumor. To Shaft by MRI BraTS2020 in 3-D data sets up preprocessing algorithm, we significantly reduced dimensions and calculated the cost of the data, from the size of each section of 155 x 240 x 240 x 4 reduced to 23*128*128*1. In this study, the input data consisted of 23 slices per sample, with each slice having dimensions of 240x128 pixels. Only the FLAIR modality was used, with a single modality employed for the analysis.

To accelerate model training and improve convergence, we implemented the Adam Optimization Algorithm (AOA) alongside dynamic learning rate adjustments. Adam, an acronym for Adaptive Moment Estimation, is a widely utilized optimization technique in training artificial neural networks. It extends the stochastic gradient descent (SGD) method, renowned for its effectiveness in handling intricate optimization challenges and extensive datasets. Adam operates by adaptively computing learning rates for each parameter, leveraging past gradients and squared gradients. It amalgamates the strengths of AdaGrad and RMSprop, transcending their limitations to deliver swift and stable convergence during training. Here’s a simplified breakdown of the AOA process: (i) Initialization: Adam sets up two moving average variables, ’m’ and ’v’, for each parameter, starting them at zero. (ii) Gradient Computation: During each training iteration, model parameter gradients relative to the loss function are calculated via backpropagation. (iii) Moving Average Update: The moving averages ’m’ and ’v’ get updated using exponential moving averages of gradients and squared gradients correspondingly, preserving past gradient information. (iv) Bias Correction: Since the initial moving averages are set to zero, they might exhibit bias, especially in early iterations. Adam corrects this bias to achieve unbiased moving average estimates. (v) Parameter Update: Ultimately, model parameters are updated using the computed moving averages, the learning rate, and a small constant (’epsilon’) to prevent division by zero. Adam’s adaptive learning rate mechanism dynamically adjusts rates for each parameter based on historical gradient data, facilitating faster convergence and robust adaptation to neural network features and gradients. Adam stands out as a popular choice for optimization in deep learning tasks due to its efficiency, minimal hyperparameter tuning requirements, and effectiveness in handling sparse gradients and large datasets. Nonetheless, it’s essential to acknowledge that the optimal optimization algorithm may vary depending on the specific problem and architecture, with alternatives like SGD with momentum, AdaGrad, and RMSprop being viable options in different scenarios.

**Fig 6:**
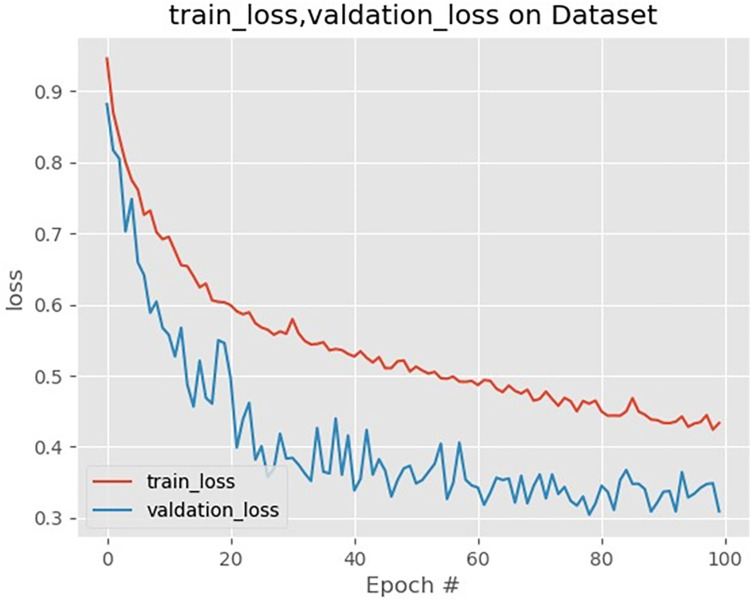
Variations of loss functions versus epoch number.

The training set, which contains ground truth labels, was split into training, validation, and test sets in an 80:10:10 ratio. The validation set assessed the model’s generalization ability. During training, the Sawin-bit-unit model utilized a custom loss function and was optimized using the ADAM optimizer with an initial learning rate of 0.0003. An adaptive learning rate strategy was implemented, decreasing the learning rate by 0.3 if the verification loss did not improve over two consecutive epochs. Further details on parameter settings can be found in Table 3, and training loss values are depicted in Fig 6.

**Table 3:** Parameter settings.

Parameters	Set values
Optimizer	ADAM
Initial learning rate	0.0003
Learning rate decay	0.3
Loss function	Dice loss
Dropout rate	0.5
Weight decay	0.0001
Batch size	23
Training periods (Epochs)	100

### 4.4. Implementation of Random Forest (RF) model

Random Forest is a widely used and effective ensemble learning technique applicable to both classification and regression tasks. It operates by generating numerous decision trees during training and then aggregating their outputs to make predictions. For classification, the final output is determined by the majority vote from all the trees, while for regression, it is the average of the individual tree predictions. The core concept behind Random Forest is to build a "forest" of decision trees, each trained on a distinct random subset of the data through a method known as bootstrap aggregating or bagging [28,29]. This means each tree is created from a sample drawn with replacement, so some data points may appear multiple times in a single tree’s training set, while others may be omitted. To make predictions, Random Forest combines the outputs from all the trees. In classification tasks, each tree casts a vote for a class, and the class with the most votes is selected as the final prediction. Optimizing a Random Forest model involves setting several important parameters. The number of trees (n_estimators) determines how many decision trees are included in the forest. Increasing this number usually enhances the model’s performance by reducing variance and preventing overfitting, but it also raises computational demands and memory usage. For instance, setting n_estimators to 300 improved the model’s accuracy from a default of 0.891 to 0.9200, showcasing the effectiveness of Random Forests in handling complex datasets. The maximum depth of the trees (max_depth) specifies how deep each tree can grow, while the minimum number of samples needed to split an internal node (min_samples_split) controls the number of samples required for a node to be split. In this study, min_samples_split was set to 6. Additionally, the minimum number of samples required at a leaf node (min_samples_leaf) determines the smallest number of samples that a leaf node can contain. This parameter helps prevent overfitting by ensuring leaf nodes have a minimum number of samples. In this study, min_samples_leaf was set to 5.

### 4.5. Implementation of Support Vector Machine (SVM) model

A Support Vector Machine (SVM) represents a robust and adaptable machine learning technique predominantly utilized for classification purposes, although it is also applicable to regression tasks. The core principle of an SVM is to identify a hyperplane that effectively distinguishes between various classes within the feature space. This hyperplane is selected to optimize the margin, defined as the distance between the hyperplane and the closest data points from each class. These nearest data points are referred to as support vectors, as they play a pivotal role in determining the hyperplane’s position and orientation. In a two-dimensional context, the hyperplane manifests as a line that segments the plane into two distinct regions, each representing a different class [28,30]. SVMs demonstrate particular efficacy in high-dimensional environments, rendering them appropriate for scenarios where the feature count significantly exceeds the number of data instances. Commonly employed kernel functions include the polynomial kernel, which transforms the data into a higher-dimensional polynomial feature space, and the radial basis function (RBF) kernel, which projects the data into an infinite-dimensional space, effectively addressing intricate relationships among features. The selection of an appropriate kernel function is vital and is contingent upon the characteristics of the data and the specific challenges being tackled. The SVM methodology entails resolving a quadratic optimization problem to ascertain the optimal hyperplane. This optimization is governed by a cost function that imposes penalties on misclassified instances while striving to maximize the margin. The resolution is achieved through convex optimization techniques, ensuring that the identified hyperplane possesses the maximum margin. In the context of the SVM model, the Radial Basis Function (RBF) emerged as the most effective kernel function during the testing phase, achieving a Kappa Coefficient (KC) of 87.22 and an F1 score of 94.13, as illustrated in Table 4. This performance was superior to that of other kernel functions when evaluated based on the F1 test score and Kappa Coefficient. Analysis of both training and testing phases revealed that the SVM-Polynomial kernel attained the second highest accuracy (KC = 72.45 and F1 = 90.01), followed by SVM-Sigmoid (KC = 70.99 and F1 = 91.58) and SVM-Linear (KC = 69.53 and F1 = 90.52), which ranked third and fourth, respectively. Furthermore, the study identified an optimal penalty coefficient (C) of 2.65, with the maximum iteration count (max_iter) established at 750.

**Table 4:** Performance of SVM model for various kernel functions.

Training Stage Testing Stage					
SVM models	Hyper-Parameters	KC	F1	KC	F1
SVM-Linear	-	75.69	94.01	69.53	90.52
SVM-RBF	Gamma = 0.01	90.25	92.56	87.22	94.13
SVM-Sigmoid	Gamma = 0.095 Coefficient = 1.05	72.96	89.17	70.99	91.58
SVM-Polynomial	Gamma = 0.65 Coefficient = 0.964 Degree = 3	78.85	90.5	72.45	90.01

### 4.6. Implementation of Extreme Gradient Boosting (XGBoost) model

XGBoost (Extreme Gradient Boosting) is a sophisticated machine learning algorithm designed to handle both classification and regression tasks. It operates within the gradient boosting framework, a technique where an ensemble of decision trees is built sequentially. Each new tree corrects the errors made by the previously trained trees, aiming to minimize a specified loss function and improve predictive performance. The algorithm builds models through boosting, which combines the outputs of several weak learners—typically decision trees—to create a strong predictive model. In each iteration, a new decision tree is added to correct the residual errors of the ensemble of previously built trees [31]. This iterative approach continues until the specified number of trees is reached or other stopping criteria are met. One of XGBoost’s standout features is its use of regularization. It includes terms for both L1 and L2 regularization in the objective function, which helps prevent overfitting by penalizing overly complex models. This built-in regularization makes XGBoost more robust to noisy data and complex patterns compared to other boosting methods. The core parameters used to configure XGBoost include number of estimators, learning rate, maximum depth, and subsample. The range of estimators varied from 20 to 100 {20,30,40,50,60,70,80,90,100}, while the learning rate values increased from 0.1 to 1 {0.1,0.2,0.3,0.4,0.5,0.6,0.7,0.8,0.9, 1}. In the XGBoost model, a limit depth of 7 was set for individual trees, and a regularization parameter of 1.5 was utilized across all iterations. The most optimal performance of the XGBoost model (F1 = 90.11 and 95.32 for the training and testing stages, respectively and KC = 91.25 and 95.17) was achieved with 80 estimators and a learning rate of 0.4.

### 4.7. Implementation of Categorical Boosting (CatBoost) model

CatBoost is a powerful and versatile machine learning algorithm developed by Yandex, designed to handle categorical features efficiently and deliver high performance across various types of predictive tasks. It is based on gradient boosting, a technique where a sequence of models, typically decision trees, are built iteratively to correct the errors of their predecessors. CatBoost leverages a gradient boosting framework that builds trees in a sequential manner, with each tree aimed at minimizing the residual errors from the previous trees. It incorporates advanced techniques to enhance model performance and reduce computational complexity, including symmetric trees, ordered boosting, and efficient handling of categorical features. This technique not only simplifies data preprocessing but also often leads to better performance by capturing the relationships between categories and the target variable more effectively. CatBoost model uses several key parameters to control its behavior and optimize its performance [32]. These parameters are designed to tune various aspects of the model, including the structure of the trees, the learning process, and the handling of categorical data. The CatBoost model was developed by taking into account three key factors: the regularization, the number of estimators (or predictors), and the values of the learning rate. The range of estimators varied from 45 to 220 {45,85,125,165,205}, while the learning rate values increased from 0.2 to 1 {0.2,0.3,0.4,0.5,0.6,0.7,0.8,0.9}. In the CatBoost model, the maximum depth of individual trees was set at 5, and the regularization parameter was fixed at 4.5 for all iterations. The most optimal performance of the CatBoost model (F1 = 89.89 and 94.35 for the training and testing stages, respectively and KC = 89.11 and 94.11) was achieved with 350 estimators and a learning rate of 0.95.

### 4.8. Implementation of Adaptive Boosting (AdaBoost) model

The AdaBoost method, also known as adaptive boosting, was introduced by Freund and Schapire [33] as an iterative ensemble machine learning algorithm employing boosting. The core concept of AdaBoost is to iteratively train multiple learners (weak learners) on the same dataset and then amalgamate their outputs to create more robust learners. The algorithm divides the sample set into multiple subsets and assigns them to the base learner for training based on their weight magnitude. The coefficients of the base learners are adjusted by calculating the error, followed by modification of the weight distribution of the sample set. Through several training iterations, all base learners are eventually combined with weights to produce a strong learner. Moreover, AdaBoost serves as a simple boosting algorithm capable of enhancing the performance of weak learners. It aims to enhance data classification accuracy by iteratively training to minimize both bias and variance. This study implemented the AdaBoost algorithm with specific parameter configurations, including 250 estimators, a learning rate of 2, the use of a linear regression loss function, and the SAMME.R (Stagewise Additive Modeling using a Multi-class Exponential loss) classification algorithm.

### 4.9. Implementation of K-Nearest Neighbors (KNN) model

The K-Nearest Neighbors (KNN) algorithm is a simple, non-parametric method utilized for both classification and regression tasks. It functions by identifying the ’k’ closest data points to a specified input, employing a distance metric such as Euclidean distance [34]. In the context of classification, the algorithm assigns the input to the class that appears most frequently among its k nearest neighbors. Conversely, for regression, it predicts the input’s value by calculating the average (or a weighted average) of the values from its k nearest neighbors. While KNN is relatively easy to comprehend and implement, it can become computationally demanding when applied to large datasets and typically necessitates feature scaling to achieve optimal performance. KNN model was derived by considering two main factors: determination of similar/closest patterns and number of neighbors. Various neighbors number ranged from 2 to 8 {2,3,4,5,6,7,8,9} and additionally the closeness of data points were assessed by various patterns (i.e., Euclidean, Manhathan, Chebyshev, and Mahalanobis). 32 KNN models were performed by Orange software. Overall, the effects of neighbors number and typical pattern detection functions on the performance of KNN models were quantified by statistical measures (i.e., F1and KC). To evaluate all performances, F1 and KC values were computed for the training and testing stages as reported in Table 5. In the case of Euclidean function, when K is equal to 4, KNN model gave the best efficacy for both training (i.e., F1 = 89.55 and KC = 94.53) and testing (i.e., F1 = 90.52 and KC = 94.32) stages in comparison with KNN models developed by other K values. By using Manhathan function, KNN model with K = 5 indicated the most promising alternative for training (F1 = 89.01 and KC = 93.94) and testing (F1 = 91.25 and KC = 93.23) stages than other KNN models. Moreover, KNN model, developed by Chebyshev function with K = 6, provided the most accurate prediction in the training (i.e., F1 = 89.50 and KC = 93.58) stage and testing (i.e., F1 = 91.41 and KC = 93.81) stage than other performances. Furthermore, in the case of Mahalanobis function, the best KNN model performance (F1 = 88.78 and 95.25 for the training and testing stages, respectively and KC = 89.85 and 93.81) were related to K = 7.

**Table 5:** KNN models performance for various setting parameters.

Training						Testing
	Distance Functions	K values	F1	KC	F1	KC
Run.1	Euclidean	2	82.96	88.25	81.39	87.23
Run.2	Euclidean	3	85.63	91.63	85.25	94.32
Run.3	Euclidean	4	89.55	94.53	90.52	94.32
Run.4	Euclidean	5	83.06	89.44	82.14	85.23
Run.5	Euclidean	6	84.32	90.36	83.25	91.88
Run.6	Euclidean	7	84.07	91.31	85.63	92.77
Run.7	Euclidean	8	83.74	91.78	84.25	90.55
Run.8	Euclidean	9	82.99	92.36	79.98	91.44
Run.9	Manhathan	2	82.50	88.33	83.41	89.32
Run.10	Manhathan	3	83.53	91.63	84.52	90.18
Run.11	Manhathan	4	86.78	92.13	87.36	91.17
Run.12	Manhathan	5	89.01	93.94	91.25	93.23
Run.13	Manhathan	6	87.05	92.99	90.14	89.32
Run.14	Manhathan	7	86.36	90.55	89.58	90.18
Run.15	Manhathan	8	85.82	91.31	91.69	91.17
Run.16	Manhathan	9	87.35	93.04	90.75	90.29
Run.17	Chebyshev	2	83.05	89.03	82.14	87.23
Run.18	Chebyshev	3	85.35	90.36	85.29	89.84
Run.19	Chebyshev	4	86.11	92.31	86.55	90.75
Run.20	Chebyshev	5	88.10	93.01	89.58	92.89
Run.21	Chebyshev	6	89.50	93.58	91.41	93.81
Run.22	Chebyshev	7	87.63	91.25	88.85	91.81
Run.23	Chebyshev	8	86.85	92.13	90.19	92.71
Run.24	Chebyshev	9	88.50	92.98	92.57	91.92
Run.25	Mahalanobis	2	83.15	85.77	84.14	88.29
Run.26	Mahalanobis	3	84.63	87.39	83.29	89.82
Run.27	Mahalanobis	4	86.89	90.65	87.99	90.71
Run.28	Mahalanobis	5	86.10	91.49	92.35	92.32
Run.29	Mahalanobis	6	88.55	93.45	88.41	92.66
Run.30	Mahalanobis	7	88.78	95.25	89.85	93.81
Run.31	Mahalanobis	8	87.32	94.13	84.11	92.78
Run.32	Mahalanobis	9	86.53	92.40	86.57	91.98

### 4.10. Evaluation metrics

In this section, various statistical measures have been used to evaluate performance of ML models [35–40]:

- The mean intersection over union (mIoU) serves as the primary metric for evaluating semantic segmentation. This measure involves computing the ratio of intersection to union for two distinct sets. The mIoU is particularly beneficial for classification tasks, as it accounts for the spatial overlap between the predicted regions and the actual ground truth, rather than merely tallying the number of correctly identified pixels. A higher mIoU score reflects superior segmentation performance, with a perfect score of 1.0 indicating flawless classification. In contrast, a lower mIoU value signifies poorer segmentation accuracy. To derive the overall mIoU, one must calculate the IoU for each individual class and then compute the average.
$$MIoU=\frac{1}{N+1}\sum_{i=0}^{N}\frac{TP}{FN+FP+TP}$$ (24)
  Initially, determine the intersection and union ratios for each category, followed by the computation of their average. True Positives (TP) refer to the positive samples that have been accurately classified, while True Negatives (TN) denote the positive samples that have been incorrectly classified. False Positives (FP) represent the negative samples that have been misclassified. TP can be conceptualized as the overlap between predicted outcomes and actual labels, whereas the sum of TP, TN, and FP constitutes the pre-union of the test outcomes and labels. A greater proximity of the intersection to the union indicates a higher accuracy in segmentation.
- ROC-AUC serves as a crucial metric for assessing the efficacy of classification models. It integrates insights from the Receiver Operating Characteristic (ROC) curve along with the Area Under the Curve (AUC). The ROC curve visually represents a classifier’s performance at various threshold levels, plotting the True Positive Rate (sensitivity) against the False Positive Rate (1-specificity). This graphical depiction illustrates the model’s capability to differentiate between positive and negative classes. The AUC, which quantifies the classifier’s overall performance, is determined by calculating the area beneath the ROC curve. AUC values range from 0 to 1, where a higher score reflects superior model performance. An AUC of 0.5 indicates a lack of discriminative power, akin to random guessing, whereas an AUC of 1.0 denotes flawless classification.
- The F1 score serves as a crucial evaluation metric that integrates both accuracy and recall, offering a holistic view of model performance. Consequently, future research will incorporate a broader range of evaluation metrics, including the F1 score and Kappa Coefficient, to enhance the validation and comparison of various data mining techniques. This approach will facilitate a more precise understanding of the strengths and weaknesses of different algorithms, thereby providing a more scientific and rational foundation for decision-making in projects.
$$\mathrm{Precision}=\frac{TP}{TP+FP}$$ (25)
$$\mathrm{Rcall}=\frac{TP}{TP+FN}$$ (26)
$$F1=\frac{2\times \mathrm{precision}\cdot \mathrm{Rcall}}{\mathrm{precision}+\mathrm{Rcall}}$$ (27)
  The F1 score is an essential metric for evaluation, as it combines both accuracy and recall, thus providing a comprehensive assessment of model performance. As a result, forthcoming research will expand the array of evaluation metrics utilized, incorporating the F1 score alongside the Kappa Coefficient to improve the validation and comparison of diverse data mining methodologies. This strategy will enable a clearer comprehension of the advantages and limitations of various algorithms, ultimately offering a more empirical and logical basis for decision-making in project contexts.
$$Kappa\;Coefficient=\frac{P_{O}-P_{e}}{1-P_{e}}$$ (28)
  where P_e_ is the probability of stochastic consistency, P_o_ is the probability of observer consistency.
- The DICE coefficient and Hausdorff distance are commonly used metrics in medical image segmentation research. The DICE coefficient measures the similarity between predicted and true segmentation maps by calculating the overlap proportion.

$$DICE=\frac{2|Y\cap \hat{Y}|}{\left|Y|+|\hat{Y}\right|}$$ (29)

where Y is the Ground Truth segmentation map, but the model predicted the segmentation map and represents the number of elements in the set. $\hat{Y}|.|$ The DICE coefficient values range from 0 (indicating no overlap) to 1 (indicating complete overlap), with higher DICE values indicating better segmentation performance.

The Hausdorff distance is a metric that measures the maximum surface distance between two segmentation maps, offering a precise evaluation of segmentation quality. It is calculated as the maximum distance between a point in each set and the closest point in the other set:

$$HD=\max\left({max}_{y\in \hat{Y}}{min}_{y\in \hat{Y}}{\left\|y-\hat{y}\right\|}_{2},{max}_{\hat{y}\in \hat{Y}}{min}_{y\in Y}{\left\|y-\hat{y}\right\|}_{2}\right)$$ (30)

*where* ‖.‖_2_ denotes the Euclidean distance. A Hausdorff distance of 0 means that the two segmentation maps are identical; however, a large Hausdorff value means that there is a large difference between the segmentations.

## 5. Results and discussions

### 5.1. Statistical performance of machine learning models

**Fig 7:**
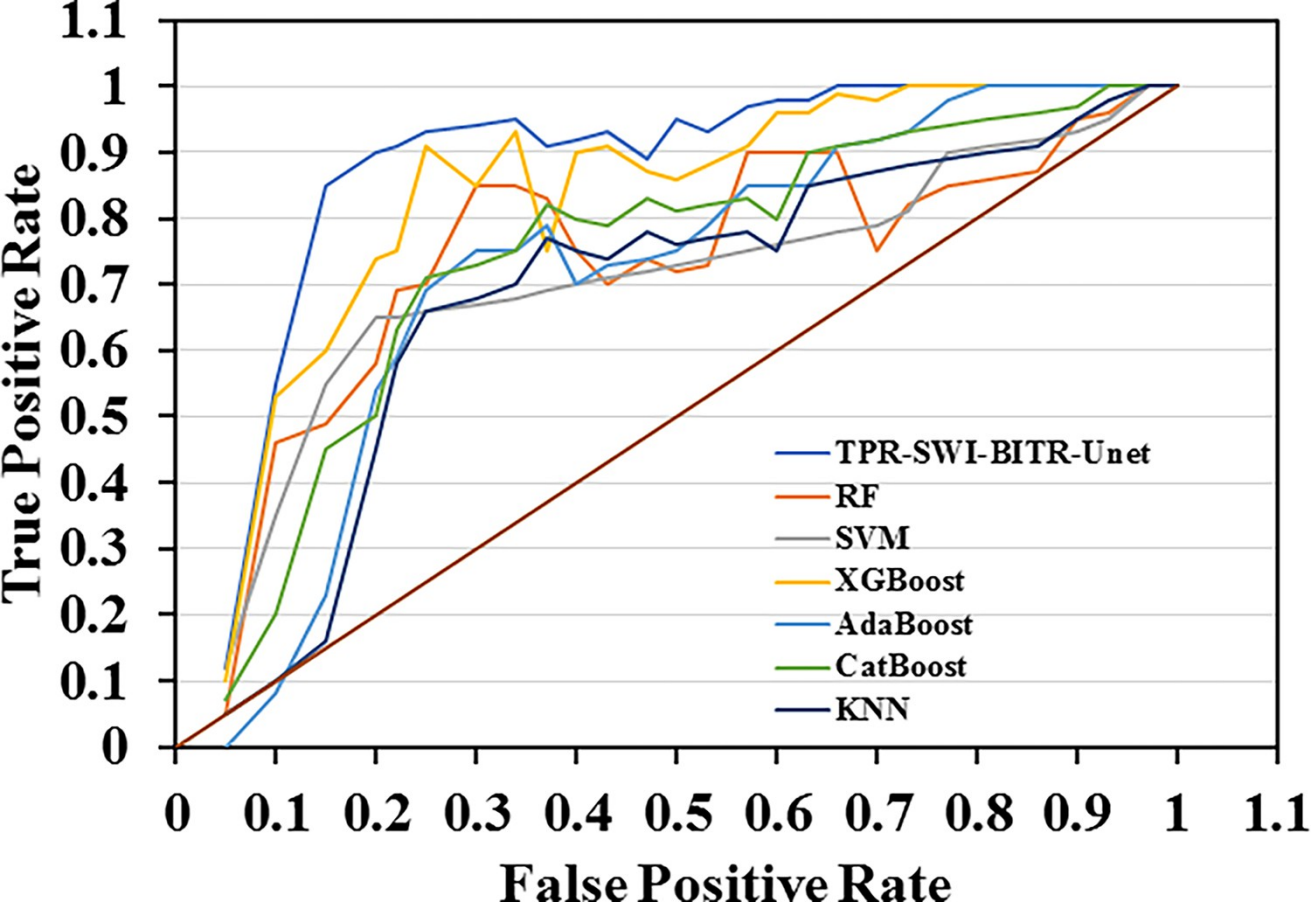
ROC-AUC plot comparison of seven ML models.

Fig 7, ROC-AUC plots were provided for ML models (i.e., SWI-BITR-UNet, SVM, RF, KNN, XGBoost, AdaBoost, and CatBoost). As a qualitative comparison on Fig 7, it was seen that RF model had the best performance in the classification task (student behavior). According to mIoU and AUC values, SWI-BITR-UNet model (AUC = 91.85 and mIoU = 0.94) indicated most promising efficacy and then followed by XGBoost (AUC = 90.86 and mIoU = 0.932), AdaBoost (AUC = 89.56 and mIoU = 0.930), CatBoost (AUC = 89.15 and mIoU = 0.926), KNN (AUC = 89.15 and mIoU = 0.918), RF (AUC = 88.51 and mIoU = 0.907), and SVM-RBF (AUC = 86.15 and mIoU = 0.893). Datasets for providing ROC-AUC curves are given in S1 Table (see Supplemental Information).

In Table 6, the statistical performance of ML models and regression model in terms of Recall, Precision, KC, and F1 were provided. From Table 6, it was found that all predictive models gave F1, Recall (Rec), and Precision (Pre) indices over 0.85, indicating satisfying performance for all ML models. According to Rec and Pre, SWI-BITR-Unet model stood at the highest level of accuracy (Rec = 97.42 and Pre = 98.11) than other ML models (e.g., Rec = 96.11, 95, 94.40 and Pre = 96.85, 95.69, 94.19 for XGBoost, AdaBoost, and CatBoost models, respectively). Similarly, values of F1 and KC proved the superiority of SWI-BITR-Unet model (F1 = 97.63 and KC = 82.25) over other ML models. Additionally, it can be said that XGBoost (F1 = 97.01 and KC = 82.01) has stood at the second level of accuracy and followed by AdaBoost (F1 = 96.13 and KC = 78.77), CatBoost (F1 = 95.31 and KC = 73.57), KNN (F1 = 88.77 and KC = 72.75), RF (F1 = 94.25 and KC = 72.35), and SVM (F1 = 921.74 and KC = 71.89).

**Table 6:** Statistical performance of ML models.

Machine Learning Models	Recall (Rec)	Precision (Pre)	KC (%)	F1 (%)	AUC (%)	mIoU
SWI-BITR-UNet	97.42	98.11	82.25	97.63	91.85	0.94
RF	93.12	93.55	72.35	94.25	88.51	0.907
SVM	92.19	92.66	71.89	91.74	86.15	0.893
AdaBoost	95.00	95.69	78.77	96.13	89.56	0.930
XGBoost	96.11	96.85	82.01	97.01	90.86	0.932
CatBoost	94.40	94.19	73.57	95.31	89.15	0.926
KNN	93.25	94.19	72.75	94.65	88.77	0.918

Extremely positive experimental results were observed in the tests performed on the Sin-BitR-UNet model. As detailed in Table 7, on the independent test set, the model performs well on the brain tumor segmentation task, especially in distinguishing between tumor core (TC) and enhanced tumor (ET), with DICE coefficients of 0.9164 and 0.9591, respectively, which are well above the current field average and then it shows that the model has high accuracy in the overlap between the predicted segmentation map and the actual annotation. In addition, the average Hausdorff distance of the model is only 1.2485mm (for TC) and 0.8242mm (for ET), which indicates that the model also achieves state-of-the-art segmentation accuracy in comparison with other ML models for example XGBoost (TC = 1.2547 mm and ET = 0.8236mm for DIC = 0.9065 and 0.9436, respectively), AdaBoost (TC = 1.2710mm and ET = 0.8165mm for DIC = 0.8965 and 0.9411, respectively), CatBoost (TC = 1.2987mm and ET = 0.8195mm for DIC = 0.8901 and 0.9348, respectively).

**Table 7:** Statistical performance of ML models on account of DIC measure.

Machine Learning Models	TC (mm)	ET(mm)	DIC1	DIC2
SWI-BITR-UNet	1.2485	0.8242	0.9164	0.9591
RF	1.2196	0.8220	0.8754	0.9309
SVM	1.2640	0.8227	0.8701	0.9325
AdaBoost	1.2710	0.8165	0.8965	0.9411
XGBoost	1.2547	0.8236	0.9065	0.9436
CatBoost	1.2987	0.8195	0.8901	0.9348
KNN	1.2113	0.8209	0.8814	0.9305

Even in the worst case, the surface distance between the predicted segmentation and the actual segmentation is very small. Further analyzing the segmentation performance of the model on different MRI modality images, we found that the model maintained a high degree of consistency and accuracy on all modalities. In particular, the DICE coefficient of the model on the FLAIR modality reaches 0.92, and the DICE coefficient on the T1 and T2 modalities also reaches 0.89 and 0.88, respectively. This finding proves that the Savin-BitR-UNet model has good adaptability when dealing with images acquired by different imaging techniques.

In evaluating stroke sequelae and monitoring rehabilitation effects, different machine learning models offer various advantages and limitations, each contributing uniquely to the task. SVM are well-regarded for their ability to manage high-dimensional spaces and find a clear margin of separation between classes. They are effective in cases where the distinction between stroke sequelae and healthy states is well-defined. However, SVMs can become computationally intensive with larger datasets and require meticulous parameter tuning, which may affect their scalability and performance on noisy data. RF provides robustness against overfitting by aggregating the predictions of multiple decision trees. This ensemble approach is beneficial for handling complex and high-dimensional datasets, such as those involved in stroke analysis. RF also delivers insights into feature importance, aiding in understanding which variables are most relevant. Despite these strengths, RF models can be less interpretable due to their complexity and may have longer prediction times with a large number of trees. AdaBoost, known for enhancing the performance of weaker classifiers, excels in improving model accuracy, particularly on imbalanced datasets where certain stroke sequelae might be less prevalent. It focuses on correcting errors made by previous models, which can be advantageous in refining predictions. However, AdaBoost’s sensitivity to noise and outliers can lead to overfitting, necessitating careful tuning and validation.

Additionally, XGBoost, a powerful gradient boosting algorithm, is highly efficient with large datasets and is renowned for its predictive accuracy. It offers extensive flexibility with hyperparameters and includes regularization techniques to combat overfitting. While XGBoost provides exceptional performance for this study (Table 8), its complexity requires careful parameter tuning and significant computational resources, making it less accessible for less experienced practitioners. CatBoost stands out for its effective handling of categorical features, which can be particularly useful in medical datasets with diverse attribute types. It often requires less parameter tuning compared to other boosting methods and is robust against overfitting. Despite its advantages, CatBoost’s complexity can demand substantial computational resources, although it generally offers a good balance between performance and ease of use. KNN model operates on the principle of classifying data points based on their proximity to others, making it straightforward and intuitive. This simplicity can be beneficial for specific tasks involving clearly clustered data. However, KNN is computationally expensive, especially with large datasets and high-dimensional features, and its performance heavily depends on the choice of distance metric and the number of neighbors. The SWI-BITR-UNet model, a specialized deep-learning approach for image segmentation, is particularly effective for tasks involving medical imaging. This application, it can accurately segment stroke-related features from MRI or CT scans, offering detailed insights into stroke sequelae. While the SWI-BITR-UNet model provides advanced capabilities in processing complex image data, it requires large datasets for training and substantial computational power, which can be a limitation for some applications. Overall, the choice of model depends on the specific nature of the data and the task at hand. Traditional models like SVM, RF, and AdaBoost models are suitable for structured data, while ensemble methods like XGBoost and CatBoost handle larger datasets and provide robust performance. SWI-BITR-UNet excels in processing and analyzing complex imaging data, making them ideal for detailed stroke evaluations. Combining different approaches or models can also enhance overall performance and provide a more comprehensive analysis.

**Table 8:** Quantification of experimental results versus related research works.

Model	Dice			Hausdorff		
	NTC	ED	ET	NTC	ED	ET
SWI-BITR-UNet	0.967	0.9591	0.9164	0.6526	1.2485	0.8242
dResU-Net [41]	0.8357	-	0.8004	-	-	
SA-Net [42]	0.8433	-	0.8177	17.9697	-	13.4298
Lightweight [43]	0.9460	-	0.8860	9.2420	-	3.4640
SEDNet	0.9308	0.9451	0.9026	0.7040	1.2866	0.7762
SEDNetX	0.9336	0.9478	0.9061	0.6983	1.2691	0.7711

**Fig 8:**
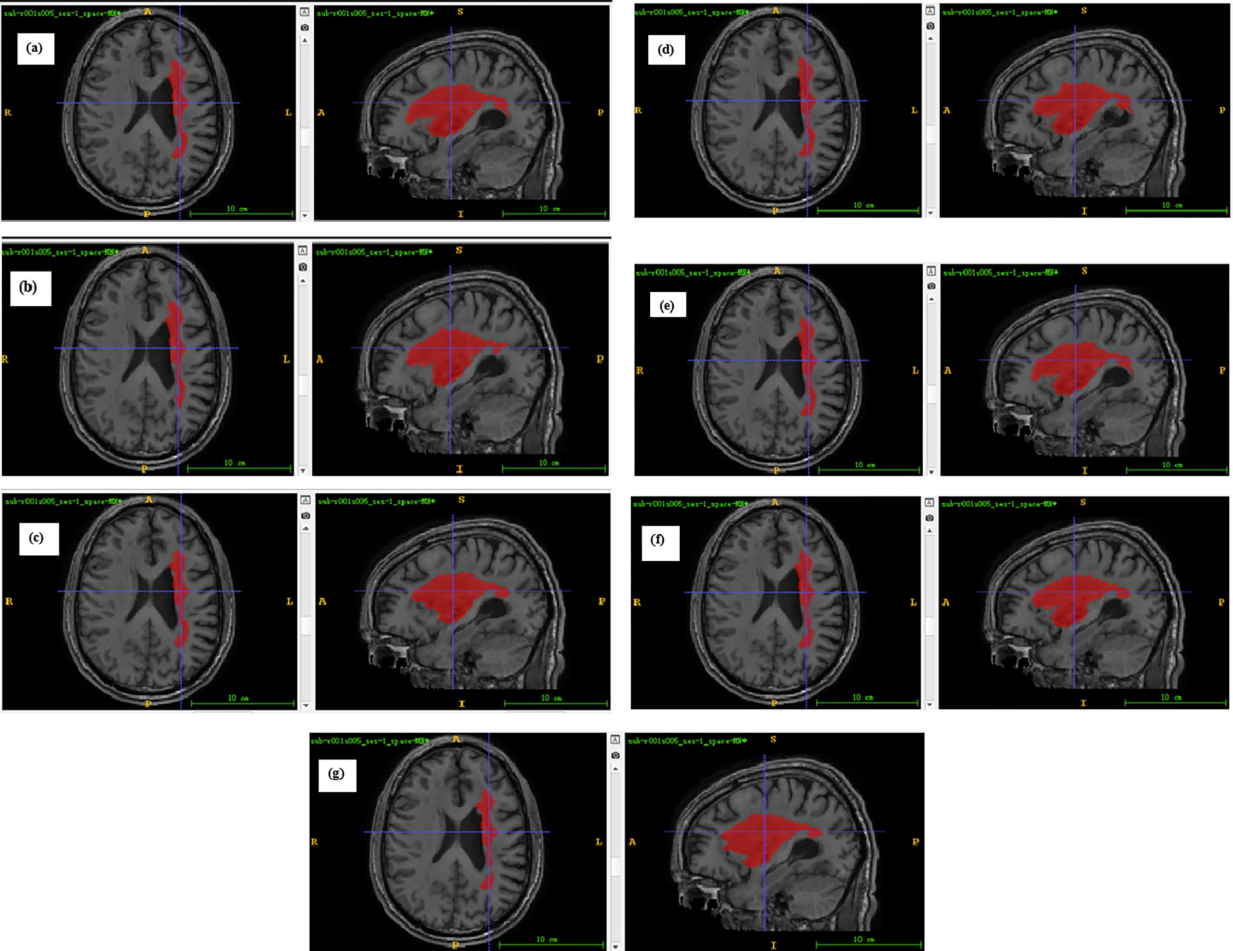
Annotated maps provided by (a) SWI-BITR-Unet, (b) XGBoost, (c) AdaBoost, (d) CatBoost, (e) KNN, (f) SVM, and (g) RF.

**Fig 9:**
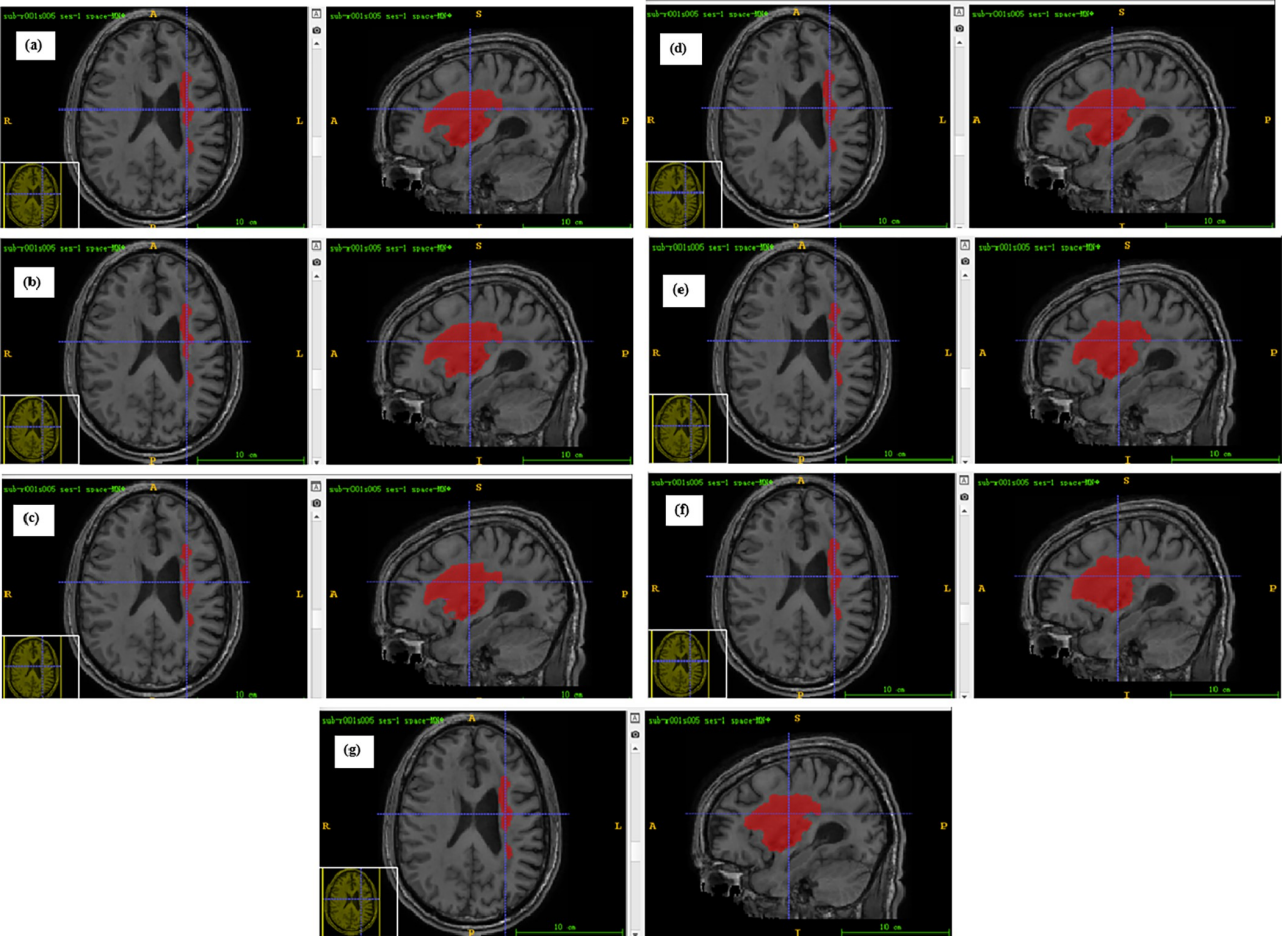
Prediction plots provided by (a) SWI-BITR-Unet, (b) XGBoost, (c) AdaBoost, (d) CatBoost, (e) KNN, (f) SVM, and (g) RF.

Annotated maps of brain tumors created by machine learning models are a powerful tool in medical imaging and oncology. These maps help in visualizing and understanding the spatial distribution and characteristics of tumors within the brain, aiding in diagnosis, treatment planning, and monitoring. The segmentation effects of the ML models through a set of images were illustrated in Figs 8 & 9. These images include Ground Truth segmentation maps, that is, segmentation results manually annotated by experts, which serve as a benchmark for us to evaluate the performance of the model. This is followed by the segmentation map predicted by the SWI-BITR-UNet, which reveals the ability of the model to automatically identify regions such as tumor core (TC) and enhance tumor (ET).

In practical clinical settings, doctors can use the precise segmentation maps generated by the SWI-BITR-UNet model to better understand the location, size, and morphology of tumors, thereby providing patients with more personalized treatment plans. In addition, the consistency and accuracy of the model across different MRI modalities indicate that it can work stably under various imaging conditions, which is of great significance for promoting the application of the model in different medical institutions. Our model’s performance on Hausdorff distance (TC of 1.2485mm, ET of 0.8242mm) further emphasizes its potential in practical applications. A smaller value of Hausdorff distance means that the predicted segmentation boundary of the model is very close to the actual boundary, which is crucial for precise tumor resection or avoiding damage to surrounding healthy tissues. In addition, compared with other machine learning models, the SWI-BITR UNet model performs better on multiple evaluation metrics, indicating that deep learning techniques have significant advantages in processing complex medical imaging data. This advantage is not only reflected in the technical aspect, but also in improving the quality of clinical decision-making and patient treatment outcomes.

The practical implications of the model’s ability to automate lesion segmentation are profound. In hospitals or clinics with limited access to specialists, the SWI-BITR-UNet model can serve as a valuable decision-support tool, assisting radiologists, and clinicians in making more informed diagnoses. It provides real-time, reliable analysis of MRI scans, helping to identify areas of brain damage or tumor growth that may be difficult to detect with the naked eye or with less advanced imaging methods. This would enable earlier diagnosis, timely intervention, and more precise monitoring of treatment progress, which are critical factors for improving patient outcomes, particularly in cases of brain tumors or post-stroke rehabilitation. Additionally, the model’s integration of advanced machine learning techniques, such as the SWIN Transformer’s local receptive field and shift mechanism, enhances its ability to capture fine-grained spatial features in 3D MRI scans. This is especially important in detecting subtle changes in brain tissue that might be indicative of early-stage stroke sequelae or tumor progression**,** where traditional imaging techniques may fall short. In practice, this means that clinicians can monitor disease progression with greater accuracy, potentially allowing for more personalized and adaptive treatment plans that are tailored to the patient’s evolving condition.

One of the most crucial contributions of the SWI-BITR-UNet model is its potential in the rehabilitation monitoring process**.** Stroke survivors, for instance, undergo long-term rehabilitation, and tracking their recovery is a complex task. Traditionally, this requires regular imaging studies, often analyzed manually, to evaluate improvements or setbacks. The ability of SWI-BITR-UNet to detect even subtle changes in brain injury areas can assist in the creation of personalized rehabilitation plans**.** For example, by monitoring the healing of specific brain regions over time, clinicians could tailor rehabilitation exercises to target specific impairments, leading to more efficient and effective therapy. Moreover, having an automated system that consistently tracks recovery may reduce the need for frequent expert evaluations, freeing up clinical time and resources while ensuring that patients still receive high-quality care.

However, while the model demonstrates strong performance, it is also essential to interpret its results with an understanding of real-world constraints**.** The reliance on large, high-quality datasets for training, such as the Bra2020 dataset**,** means that its performance might be optimized for data that closely resembles the training set. In clinical practice, however, variability in MRI scans from different institutions or scanners could pose a challenge, especially when the model encounters data with varying quality or resolution. Although the study presents the SWI-BITR-UNet as a promising model, further research will be required to ensure that it can maintain its accuracy and reliability across diverse real-world clinical data. This could involve integrating the model into multi-center studies or incorporating domain adaptation techniques to ensure that it performs well across diverse imaging conditions. Another consideration is the model’s computational demands**.** While the results show that the model is highly accurate, its complex architecture—particularly the use of 3D CNNs, transformers, and attention mechanisms—requires significant computational resources. This may limit the feasibility of deploying the model in resource-constrained environments where high-performance computing systems are not available. In clinical practice, especially in remote or underserved areas, the ability to provide real-time analysis on standard hardware or mobile devices is critical. Addressing this issue through model optimization techniques, such as pruning, quantization, or developing lighter versions of the model, will be essential for practical deployment in diverse clinical settings. Finally, the interpretability of the model remains a crucial factor for its adoption in clinical practice. While the model’s ability to perform highly accurate segmentation is a key strength, clinicians will need transparency to trust its predictions fully. The lack of explainability in deep learning models can hinder their acceptance, as medical professionals need to understand the rationale behind the model’s decision-making process. The integration of explainable AI techniques—such as attention maps or saliency maps—will help to elucidate how the model identifies brain lesions, making it easier for clinicians to understand its outputs and integrate them into their clinical decisions confidently.

In summary, the findings of this study underscore the promising potential of the SWI-BITR-UNet model for advancing neuroimaging in both stroke and brain tumor rehabilitation contexts. The model’s superior segmentation accuracy and ability to monitor changes in brain lesions over time offer substantial benefits for early diagnosis, personalized treatment planning, and rehabilitation. However, challenges remain, particularly in ensuring generalization across different datasets, optimizing for real-time use in clinical settings, and increasing interpretability. By addressing these issues, future research can further refine the model’s applicability, ultimately leading to more efficient and effective clinical decision-making.

### 5.2. Comparisons with literature

Compared with other existing models, such as dResU-Net, SA-Net, and Lightweight model, as well as SEDNet and SEDNetX, Samin-BitR-UNet shows obvious advantages in DICE coefficient, especially in distinguishing tumor core (TC) and enhanced tumor (ET). Moreover, teachers BiTr—Unet also outperform on the Hausdorff distance, or at least can be comparable to these models, which further proves the teachers BiTr-Unet on brain tumor segmentation task performance excellence. Table 8 indicated the comparisons of the present results with related research works. Compared with the present study, in [41], the proposed architecture yielded encouraging outcomes, with the average dice scores for tumor core (TC), whole tumor (WT), and enhancing tumor (ET) on the BraTS 2020 dataset standing at 0.8357, 0.8660, and 0.8004, respectively. To showcase the proposed model’s robustness in real-world clinical scenarios, validation was conducted on an external cohort comprising 50 randomly selected patients from the BraTS 2021 benchmark dataset. The attained dice scores on this external cohort were 0.8400, 0.8601, and 0.8221 for TC, WT, and ET, respectively. A comparative analysis with state-of-the-art techniques indicates that dResU-Net can significantly enhance the segmentation performance of brain tumor sub-regions. Additionally, In [42], the proposed framework underwent training with the 369 challenge training cases made available by BraTS 2020, achieving an average Dice Similarity Coefficient (DSC) of 0.8828, 0.8433, and 0.8177, along with a 95% Hausdorff distance (measured in millimeters) of 5.2176, 17.9697, and 13.4298 for whole tumor, tumor core, and enhanced tumor, respectively. These results secured its position as the 3rd among 693 entries in the BraTS 2020 challenge based on testing 166 cases [42].

The method, proposed by Zhao et al.[3], has shown promising outcomes on the BRATS 2020 testing datasets as well as in the present study. Unlike other leading techniques, our approach achieves competitive performance using only three imaging modalities (Flair, T1c, and T2), rather than four (Flair, T1, T1c, and T2). During the period, T1 and T2 represent T1-weighted images and T2-weighted images. Flair stands for Magnetic Resonance Imaging Liquid Attenuation Reversal Sequence.we participated in the BRATS 2016 challenge and attained the top rank in its multi-temporal evaluation. In [43], the methodology relies on 2D Fully Convolutional Neural Networks (FCNNs) and Conditional Random Field Recurrent Neural Networks (CRF-RNN) to ensure computational efficiency. They utilized image slices as training data for CRF-RNN training and fine-tuning the integrated FCNNs and CRF-RNN. However, the imbalance in pixel numbers across different classes in image slices may potentially degrade segmentation performance [44,45].

In the process of implementing the SWI-BITR-UNet model and comparing it with other machine learning models, we encountered several challenges that are crucial for the transparency and depth of our research. Firstly, due to the complexity and diversity of medical imaging data, models require a high degree of adaptability and robustness when dealing with different modalities and patient characteristics. We enhance the feature extraction capability of the model by using multimodal MRI data and introducing CBAM attention module to address this challenge. The training of the second model requires a lot of computing resources and time, especially when processing 3D medical image data. To solve this problem, we optimized the network structure and adopted depthwise separable convolution to reduce model parameters and computational complexity.

The third issue of data imbalance is also a problem that we need to pay special attention to during the training process. The sample size of certain categories in the brain tumor dataset may be small, which may affect the generalization ability of the model. We use oversampling and undersampling techniques to balance categories and ensure that the model does not bias against the majority of classes.

In S2 Table, full descriptions of abbreviations used in this study were provided.

### 5.3. Current challenges in neuroimaging with SWI-BITR-UNet

Despite the advancements introduced by the SWI-BITR-UNet architecture, several challenges persist that need to be addressed to improve its performance and clinical applicability.

One of the primary challenges is the variability of medical imaging data. MRI scans can exhibit significant differences across patients, in terms of quality, resolution, and artifacts. This variability can make accurate segmentation difficult, particularly when lesions appear subtle or irregular in shape. Additionally, manual labeling of training datasets for lesions, while crucial for supervised learning, is a time-consuming and error-prone process that suffers from inter-observer variability. This can lead to inconsistencies in the training data, which in turn affects the model’s ability to generalize to new patients or different data sources. Another challenge is generalization across different imaging modalities and scanners. While SWI-BITR-UNet performs well on certain MRI data types, it may struggle when exposed to images from different scanners or imaging protocols. Differences in magnetic field strengths, scanner models, and acquisition settings can result in variations in image quality and resolution. These discrepancies can affect the model’s ability to perform accurate segmentation across diverse clinical settings. In practice, patients often have scans from different machines, so the model must be able to generalize well across these variations for clinical deployment. The handling of low-resolution and noisy MRI scans remains another significant challenge. MRI images can sometimes be of lower resolution or suffer from noise and motion artifacts. Low-resolution images, in particular, may not contain enough detail to enable accurate lesion detection, which is critical in clinical settings where early-stage tumors or subtle stroke damage need to be identified. While SWI-BITR-UNet can process such images, it may still struggle with such data, requiring additional techniques for noise removal and enhancement.

The complexity of the SWI-BITR-UNet model, with its combination of transformers, 3D convolutional networks, and attention mechanisms, results in high computational demands. This presents a barrier to real-time or large-scale deployment, particularly in clinical environments with limited computing resources. Training the model requires significant computing power, and even once trained, the model’s resource-intensive architecture may hinder its application in routine clinical practice where faster processing is needed. Lastly, the interpretability of the model remains a challenge. Deep learning models, including SWI-BITR-UNet, are often seen as “black boxes,” meaning it is difficult to understand the rationale behind the model’s predictions. In a clinical setting, where decisions based on the model’s output can directly impact patient care, clinicians need to trust the model’s results. Without transparency or the ability to explain why certain lesions are detected or segmented in a particular way, the model may face resistance from healthcare professionals who prefer more explainable decision-support tools.

### 5.4. Robust recommendations for further research directions

To address these challenges and further enhance the capabilities of the SWI-BITR-UNet model, several research directions are recommended.

First, to overcome the issue of data variability and the limited availability of labeled data, data augmentation and synthetic data generation methods should be explored. These approaches can artificially expand training datasets by creating variations of the existing data, which can help the model generalize better to different patients and imaging conditions. Synthetic data generation using techniques like Generative Adversarial Networks (GANs) can also supplement real-world data, especially in cases where rare lesions are present. These synthetic datasets could be useful for training the model to handle diverse lesion types and help address the lack of labeled data.

Another key area for improvement is the model’s ability to generalize across different imaging modalities and scanners. Research into multi-modal learning could be particularly beneficial, allowing the model to simultaneously process information from various imaging sources, such as PET scans, CT scans, or different MRI sequences. This would help the model adapt to the variability in imaging protocols across clinical settings. In addition, exploring domain adaptation techniques could help the model transfer its learned knowledge from one dataset or imaging modality to another, enhancing its robustness when applied to diverse clinical environments. To better handle low-resolution and noisy data, further investigation into advanced denoising and preprocessing techniques is essential. Methods such as denoising autoencoders or wavelet transforms could be employed to clean up noisy MRI scans before they are fed into the model. Enhancing the model’s ability to process low-resolution scans without sacrificing accuracy could also involve modifying its architecture to focus more on the extraction of relevant features from these challenging datasets. Given the model’s high computational demands, future research should focus on developing more efficient architectures that maintain or improve the model’s performance while reducing the computational burden. Techniques such as model pruning, quantization, and knowledge distillation could help reduce the size of the model and make it more suitable for deployment in clinical settings where real-time processing is crucial. Additionally, exploring lightweight transformer models or efficient convolutional networks tailored to medical imaging could make SWI-BITR-UNet more accessible to a broader range of clinical environments.

Another critical direction for future work is the interpretability of the model. To make the model more clinically acceptable, it is essential to provide clinicians with clear explanations for its predictions. Research into methods that generate visual explanations, such as saliency maps or attention maps, could help illuminate which parts of the image are influencing the model’s decisions. Techniques such as Grad-CAM or Layer-wise Relevance Propagation (LRP) could be incorporated to provide these explanations, making the model more transparent and enabling clinicians to better understand how the model arrived at its conclusions. Finally, integrating SWI-BITR-UNet into real-time clinical settings requires improving its real-time performance. This can be achieved by optimizing the model for use on edge devices or GPUs, which could speed up processing times and make the model feasible for point-of-care applications. Moreover, integrating the model with existing clinical workflows and electronic health record systems could help healthcare professionals use the model’s output in conjunction with other diagnostic tools, ultimately improving decision-making and patient care. In the long term, SWI-BITR-UNet could be applied to personalized medicine, where the model would play a crucial role in longitudinal monitoring of patients over time. By incorporating additional patient-specific data, such as genetic information or clinical history, the model could help create more tailored and effective treatment plans for individuals. Furthermore, multi-task learning could be explored to enable the model to predict not only lesion segmentation but also tumor growth or recovery progress, providing clinicians with a comprehensive tool for tracking patient outcomes.

By addressing these challenges and exploring these research directions, SWI-BITR-UNet could evolve into a more robust, efficient, and clinically applicable tool. These advancements will not only improve the model’s accuracy and generalization but also pave the way for more widespread adoption in real-world medical settings, ultimately improving patient outcomes.

## 6. Conclusion

This paper proposed an advanced neuroimaging technique, the Swi-BitR-UNet model, to assess stroke sequelae and monitor rehabilitation outcomes. The model combined taught local receptive field and the displacement mechanism of the Transformer and U-features fusion strategy in the.net framework, significantly improving the multimodal MRI scans of the midbrain damage area on the accuracy of the segmentation. Through the application of a 3D CNN encoder and decoder, as well as the integration of the CBAM attention module and skip connection, the model achieved a high level of segmentation accuracy, while maintaining high computational efficiency and the ability to deal with long-distance dependence. Experimental results showed that the teachers BiTr-Unet model not only could effectively identify the subtle changes in the damaged region cerebral apoplexy and monitoring, and in the rehabilitation process provided new and efficient tools. To reach more robust comparisons, various machine learning models (i.e., RF, SVM, XGBoost, AdaBoost, CatBoost, and KNN) were used to efficiently train and test the BRATS2020 dataset for tumor progress in brain. Setting parameters given by ML models were adjusted to obtain optimal results through the training and testing stages. Several statistical measures (e.g., F1, mIoU, KC, AUC) have been employed to assess the performance of ML models. In this way, SWI-BITR-Unet model demonstrated the highest level of accuracy (F1 = 97.63, mIoU = 0.94, KC = 82.25, and AUC = 91.85) when compared with other machine learning models. Additionally, in terms of qualitative comparisons, SWI-BITR-Unet model showed excellent performance, especially in differentiating Hausdorff distance characterizations (TC and ET) and DICE coefficient reached 0.9164 and 0.9591 respectively, and TC and ET obtained 1.2485 mm and 0.8242 mm. The results were completely comparable to other ML models and related research works. Furthermore, the segmentation performance of the model on images from different MRI modalities shows a high degree of consistency and accuracy, which proves its adaptability when dealing with multimodal data. Compared with other existing models, Samin-BitR-UNet shows obvious advantages in both the DICE coefficient and Hausdorff distance, which further proves its excellent performance in brain tumor segmentation tasks.

## Supporting information

S1 Table Original datasets for providing ROC-AUC curves. (XLSX)

S2 Table Full descriptions of abbreviations used in this study. (DOCX)
