# Supplementary material for: Evaluation of stroke sequelae and rehabilitation effect on brain tumor by neuroimaging technique: A comparative study
Source: PLoS One. 2025 Feb 24;20(2):e0317193. doi: 10.1371/journal.pone.0317193 (PMC11849865; doi:10.1371/journal.pone.0317193)
Supplement: S2 Table — (DOCX) [file pone.0317193.s002.docx]

| ML | Machine Learning |
| --- | --- |
| DTI | Diffusion Tensor Imaging |
| CBAM | Convolutional Block Attention Module |
| Rec | Recall |
| Pre | Precision |
| F1 | F1 Score |
| KC | Kappa Coefficient |
| mIoU | mean Intersection over Union |
| RF | Random Forest |
| SVM | Support Vector Machine |
| KNN | K-Nearest Neighbor |
| TC | Tumor Core |
| ET | Enhancing Tumor |
| ReLU | Rectified Linear Unit |
| IOU | Intersection Over Union |
| NCR | Necrotic Tumor Core |
| SGD | Stochastic Gradient Descent |
| AOA | Adam Optimization Algorithm |
| TP | True Positives |
| TN | True Negatives |
| ROC | Receiver Operating Characteristic |
| AUC | Area Under the Curve |
| F^d^_i_ | Output Feature map of layer |
| D | Depth Convolution Kernel |
| $F_{i}$ | Residual connection |
| $A^{c}$ | Output of channel attention |
| $A^{s}$ | Output of spatial attention |
| E | Embedded Feature |
| W | Learnable Weight Matrix |
| PE | Positional Encoding |
| 𝑘 | Frequency factor in location coding |
| $E_{embed}$ | Features embedded layer |
| $F_{in}$ | Input Features |
| $F_{d}$ | Depth of the output of the convolution |
| F_pw_ | Output of the point-by-point convolution |
| Q, K, V | Query, Key, Value matrix |
| MultiHead(Q,K,V) | Multi-head attention mechanism |
| $W_{i}^{Q},W_{i}^{K}, and W_{i}^{V}$ | Weight matrix for the query projection |
| F_u_ | Upsampled feature map |
| C_u_ | Number of output channels |
| K_u_ | Size of the transposed convolution kernel |
| S_u_ | Step size |
| F_r_ | Layer 3-D convolution |
| K_r_, S_r_, and P_r_ | Size of the convolution kernel, step length, and filling |
| C_skip_ | Skip connections |
| $F_{fuse}$ | Output of the corresponding layer |
| $F_{skip}$ | Characteristic of the jump connection diagram |
| $F_{encode}$ | Characteristic of the merged diagram |
